# Dealing with uncertainty: A high-density EEG investigation on how intolerance of uncertainty affects emotional predictions

**DOI:** 10.1371/journal.pone.0254045

**Published:** 2021-07-01

**Authors:** Fiorella Del Popolo Cristaldi, Giovanni Mento, Michela Sarlo, Giulia Buodo

**Affiliations:** 1 Department of General Psychology, University of Padua, Padova, Italy; 2 Padua Neuroscience Center (PNC), University of Padua, Padova, Italy; 3 Department of Communication Sciences, Humanities and International Studies, University of Urbino Carlo Bo, Urbino, Italy; Sapienza University of Rome, ITALY

## Abstract

Intolerance of uncertainty (IU) can influence emotional predictions, constructed by the brain (*generation* stage) to prearrange action (*implementation* stage), and update internal models according to incoming stimuli (*updating* stage). However, neurocomputational mechanisms by which IU affects emotional predictions are unclear. This high-density EEG study investigated if IU predicted event-related potentials (ERPs) and brain sources activity developing along the stages of emotional predictions, as a function of contextual uncertainty. Thirty-six undergraduates underwent a S1-S2 paradigm, with emotional faces and pictures as S1s and S2s, respectively. Contextual uncertainty was manipulated across three blocks, each with *100%*, *75%*, or *50%* S1-S2 emotional congruency. ERPs, brain sources and their relationship with IU scores were analyzed for each stage. IU did not affect prediction *generation*. During prediction *implementation*, higher IU predicted larger Contingent Negative Variation in the *75%* block, and lower left anterior cingulate cortex and supplementary motor area activations. During prediction *updating*, as IU increased P2 to positive S2s decreased, along with P2 and Late Positive Potential in the *75%* block, and right orbito-frontal cortex activity to emotional S2s. IU was therefore associated with altered uncertainty assessment and heightened attention deployment during *implementation*, and to uncertainty avoidance, reduced attention to safety cues and disrupted access to emotion regulation strategies during prediction *updating*.

## Introduction

The world is an uncertain place, and the uncertainty we experience in everyday life can have a dramatic influence on our emotional life. For example, the COVID-19 pandemic outbreak, and the associated risk of contracting the virus, carried a huge amount of uncertainty, substantially impacting over the emotional experience and mental health of people worldwide. Or else, not knowing how the plot is going to develop while watching a new TV series usually increases the rewarding value of the series itself. From both examples, it is clear how dealing with uncertainty can intensify affective experience.

The literature has long suggested that uncertainty pushes to a polarization of the impact of both positive and negative affect, leading to an increased attention and emotional engagement towards uncertain events [1]. Furthermore, individual differences in the subjective predisposition to tolerate uncertainty also play a non-negligible role in shaping emotional processing within uncertain environments. Intolerance of uncertainty (IU) is the dispositional characteristic that reflects individual differences in tolerating and adapting to uncertain situations [2]. It has been defined as the inability to tolerate the aversive reaction triggered by a perceived lack of sufficient or salient information, sustained by the correlated perception of uncertainty [3]. Individuals high in IU tend to appraise uncertainty as threatening irrespective of its probability and outcomes, to experience heightened physiological arousal in uncertain situations, and to consider the possibility of the occurrence of a negative event as unacceptable [2,4,5]. IU is characterized by cognitive, affective, and behavioral facets that have a broad influence on emotional experience, i.e., inflated estimates of threat probability, increased attention to threat and hypervigilance, deficient safety learning, behavioral and cognitive avoidance of exposure to uncertainty, and heightened physiological reactivity to threat uncertainty [6]. Moreover, the fear of the unknown (which is assumed to be the key feature at the basis of IU) has been proved to be a lower-order construct able to account for statistically significant variance in several higher-order constructs [2,7,8]. Indeed, it is positively associated with neuroticism [3,9], and with symptoms of different anxiety disorders (see [3] for a review); and it can act as a trigger for the activation of the behavioral inhibition system (BIS) [2,3], and the fight-flight-freeze defensive response [2,3]. For these reasons, IU has been proposed as a sound trans-diagnostic risk and maintaining factor, potentially crucial for vulnerability assessment and/or as a target for clinical treatment, for a wide range of emotional traits and disorders, such as neuroticism, trait anxiety and negative affectivity traits, as well as anxiety disorders, depressive disorders, obsessive-compulsive disorders, trauma- and stressor-related disorders, and eating disorders [1–4,10].

In the last years, the degree of uncertainty intrinsic to contextual information has been targeted as an influential feature in shaping emotional processing, as defined within the predictive coding framework [11–14]. According to this conceptualization, emotions are interoceptive predictive models, developing across three neurocomputational stages: prediction *generation*, in which the brain generates predictions according to prior experience and contextual information available in the environment; prediction *implementation*, in which bodily and behavioral changes are prearranged to efficiently deal with the predicted situation; prediction *updating*, in which predictions are compared with actual incoming stimuli, and mismatching ones are encoded as prediction errors and used to adjust future predictions [11–16]. Contextual differences in the relative balance of the amount of information between certain and uncertain situations are assumed to be crucial in determining whether uncertainty is appraised as threatening [3]. Moreover, it has been proposed that biases in the anticipation of emotional stimuli (e.g., inflated threat estimates) characterizing high-IU individuals could result from a disrupted prediction error signaling, which in turn results in a failure to update emotional predictions [6]. For these reasons and given the significance of IU as a trans-diagnostic risk factor, a better understanding of how individual differences in IU affect the neural correlates of emotional predictions as a function of contextual uncertainty could represent a valuable contribution to advancing knowledge in the field, leading to promising clinical and preventive implications. In fact, human organisms are embedded in constantly changing environments, conveying information with varying degrees of (un)certainty, and in which a continuous adaptation of emotional predictions is required. Assessing the neural correlates of individual differences in IU across the different neurocomputational stages of emotional predictions would allow to identify potential targets to treat, or to early intervene on, thus preventing high-IU from developing into clinically relevant conditions.

Nevertheless, experimental studies investigating at a neurocomputational level the effects of IU on emotional processing as a function of contextual uncertainty [17–23] are still scarce and provided conflicting results, with none of them embracing the predictive perspective on emotions [13,14]. Amongst these studies, uncertainty was manipulated mainly through two experimental paradigms. Some studies, measuring startle reflex and event-related potentials (ERPs), employed threat-of-shock paradigms [17,18,24], in which participants were presented with cues preceding predictable vs. unpredictable electric shocks. Other studies, recording skin conductance, electromyographic activity (EMG), ERPs, or functional magnetic resonance imaging (fMRI) [19–23], employed emotional S1-S2 paradigms, with 100% vs. 50% instructed emotional congruency between S1s (symbolic cues) and S2s (emotional pictures). Experimental tasks varied from passive viewing of stimuli [20,22], to tasks requiring shock/S2 ratings [17,18,21,23], or expectancy ratings about the upcoming S2 [19,21]. All these studies measured IU through the Intolerance of Uncertainty Scale (IUS), either in its original (27-item) or short (12-item) form [5,25], and divided the samples into two groups (high- vs. low-IU) according to the IUS scores distribution. Uncertainty was manipulated mostly through two extreme levels (fully predictive vs. random), and only negatively-valenced emotional stimuli were employed and compared with a neutral condition. Importantly, both S1-S2 and threat-of-shock paradigms can be reconsidered within the predictive framework [13,14] as a function of the three stages of the construction of emotional predictions, with S1/cue processing potentially reflecting the *generation* stage, the inter-stimulus interval (ISI) between S1/cue and S2/shock reflecting the *implementation* stage, and the S2/shock processing representing prediction *updating*.

However, the results collected are fragmentary and sometimes in conflict with the predictions formulated by the theoretical models of IU [1,2,4,6]. Regarding the *generation* stage, some studies [19,22] found no differences between high- and low-IU participants in S1/cue-locked skin conductance and corrugator EMG responses as a function of cue predictive meaning. Other studies [18,20] found that high-IU participants showed a larger P2 ERP component to S1s/cues irrespective of their predictive meaning, reflecting a heightened early automatic attention allocation towards cues and their potential threatening meaning. Focusing on prediction *implementation*, most of the studies found no significant effects of IU on ERPs amplitude, skin conductance, EMG responses, and expectancy ratings measured during the ISI between S1/cue and S2/shock [18,21,24]; while [19] found that high-IU participants showed higher threat expectancy ratings following cues conveying uncertain predictive information, consistently with the overestimation of threat predicted by the theoretical models of IU [6]. As for the prediction *updating* stage, some studies measuring skin conductance [21,22] failed to find significant effects of IU on S2-locked indices. Other studies found that in conditions of uncertainty IU negatively predicted neural activity within posterior frontomedian cortex (PFMC) [23], and the magnitude of the startle response [17], thus unexpectedly suggesting that as IU increases, the aversive reactions to uncertain threat attenuate, probably due to the perceived lack of controllability typical of uncertain contexts. Lastly, in high-IU participants uncertain negative S2s were found to elicit smaller Late Positive Potential (LPP) amplitudes, reflecting a dampening effect on the emotional processing of uncertain stimuli [20]. Altogether, the results related to the *updating* stage are in contrast with the assumption that uncertainty is associated with affect intensification and heightened physiological reactivity, as predicted by the theoretical models of IU [1,2,4,6].

The present study aimed to investigate through high-density EEG whether and how IU predicted the neural activity, both at the scalp and at the source level, which develops in contexts of different predictive value across the stages of *generation*, *implementation* and *updating* of emotional predictions. To this aim, we employed an emotional S1-S2 paradigm (cfr. [26]) in which contextual uncertainty was manipulated by means of valid (*100%*), moderately predictive (*75%*), and random (*50%*) emotional congruency between S1 and S2. Standardized emotional faces and pictures with positive, negative, or neutral valence were employed as S1s and S2s, respectively. Furthermore, we measured IU through the Italian adaptation of the IUS [27], and we used it in our analyses as a continuous predictor rather than dichotomizing its scores into two groups, thus avoiding flattening individual differences in IU and losing information and power [28]. Besides allowing to separately investigate the three stages of emotional predictions, the S1-S2 paradigm we employed contributed to fill at least three gaps in the extant literature. First, an intermediate level of contextual uncertainty (*75%*), which could better resemble a more realistic, moderately certain environmental context, was included. Second, a condition of positive valence was added, thus allowing to disentangle valence- vs. arousal-specific effects. Third, the optimal compromise between spatial and temporal resolution offered by high-density EEG [29] allowed to provide a compelling comprehensive neurocomputational evidence, thus contributing to narrow the gap between ERP and fMRI findings.

For each neurocomputational stage, we targeted specific ERP components, which have been shown to respond to the experimental manipulation (cfr. [26]). As for prediction *generation*, we focused on S1-N170, which is sensitive to predictive contexts, and reflects structural encoding and coarse emotional processing of facial expressions [30–32]. As to the *implementation* stage, we targeted the Contingent Negative Variation (CNV) [33] arising during the ISI, which reflects the orientation of attention towards motivationally relevant stimuli (early portion), and the motor preparation for a required or possible action (late portion), besides being sensitive to predictive contextual factors [34–37]. As regards prediction *updating*, we considered both early and late S2-ERPs, i.e., the P2, indexing early automatic attention allocation and the degree of updating of internal models, and the LPP, reflecting late motivated and sustained attention to emotional stimuli [37–41].

We hypothesized higher IU scores to predict (i) a larger N170 irrespective of the predictive context [18,20] during prediction *generation*; (ii) larger early and late CNV amplitudes following negative S1s (reflecting the anticipatory processes elicited by inflated threat estimates, see [19]) in uncertain predictive contexts (*75%* and *50%*) during the *implementation* stage; (iii) either smaller (according to ERP findings, e.g., [20]) or larger (according to the IU theories’ predictions, e.g., [1]) P2 and LPP amplitudes to emotional S2s in uncertain predictive contexts (*75%* and *50%*) during prediction *updating*.

## Methods

### Participants

Thirty-six right-handed undergraduates at University of Padova took part in the study as volunteers (16 males, age: *M* = 23.25, *SD* = 1.85, range = 20–29). The sample size was based on previous ERP research measuring IUS within S1-S2 paradigms [20]. A post-hoc power analysis (α = 0.05, η^2^ = 0.02) showed that the final sample size (N = 36) allowed to reach an average power of 0.541.

A total of 294 undergraduates were initially screened for participation through an online survey evaluating the inclusion criteria for the study: no history of neurological or psychiatric disorders; normal or corrected-to-normal vision; no medication; right-handedness, as assessed by the Edinburgh Handedness Inventory [42]; absence of extreme levels of blood-injection-injury fear, as assessed by the Fear Survey Schedule [43,44]. Since some negative pictures depicted gory scenes, highly fearful participants (N = 108), who scored 4 on items of the Fear Survey Schedule concerning blood, injuries and/or weapons (0–4 score), were discarded from participation for ethical reasons. Of the remaining participants, 23 were excluded because left-handed, and 127 because they did not meet the other inclusion criteria.

All participants signed an informed consent. All experimental procedures were approved by the Ethical Committee for the Psychological Research of the University of Padua (protocol no. 2859) and were conducted in accordance with the Declaration of Helsinki.

### Stimulus material and procedure

Before arrival, all participants answered an online version of the IUS, administered in its Italian 12-item validated adaptation [27]. Participants were instructed to answer the survey alone, in a silent room, taking all the time needed.

Upon arrival, participants seated in a dimly lit room, at a 90-cm viewing distance from a 24-inch computer monitor (1280 x 1024 resolution). They received detailed information about EEG montage and the experimental task. Then, an elastic 128-ch EEG net was applied for EEG recording.

After a 10 min adaptation period, a computerized S1-S2 paradigm was presented on the screen. S1s were 24 colored emotional faces selected from the NimStim Set of Facial Expressions [45]: 4 male and 4 female Caucasian models, each posing a fearful, happy, and neutral facial expression. S2s were 120 colored emotional pictures selected from the International Affective Picture System (IAPS) [46]: 40 highly-arousing negative, 40 highly-arousing positive, and 40 neutral. Positive and negative emotional pictures did not differ for mean standardized arousal ratings (*Ms* = 6.43 and 6.44, respectively; *t*(93) = 0.12, *p* = .9). NimStim and IAPS picture numbers are listed in S1 File. S1s and S2s were presented pseudo-randomly (no more than 3 subsequent same-valence couplings) in their original size, in the center of the screen against a black background, through E-prime software [47].

At the beginning of the S1-S2 paradigm, participants read the on-screen the task instructions at their own pace. They were told that they would see a face followed by a picture and that they only had to look at the screen, trying to move as little as possible. Each trial started with the presentation of S1 for 500 ms, followed by a fixed inter-stimulus interval (ISI) of 2000 ms, in which the screen remained black. Then S2 was presented for 1500 ms. A white fixation cross against a black background was displayed during the inter-trial interval (ITI), whose length randomly varied between 800 and 1200 ms. The total number of trials was 360.

S1 and S2 *valence* was manipulated within-subjects through three levels: *positive* (POS), *negative* (NEG) and *neutral* (NEU). Contextual *uncertainty* was implicitly manipulated within-subjects through 3 continuous blocks of 120 trials each. In the *100% block*, S1-S2 emotional congruency was complied in all the trials, that is, S1 valence was fully predictive of S2 valence. In the *75% block*, S1-S2 pairs were congruent in 75% of the trials, so S1 valence was moderately predictive of S2 valence. In the *50% block*, S1 valence was unpredictive of S2 valence, since each S1 could be randomly followed by a positive, negative, or neutral S2. The blocks seamlessly followed one another, with brief breaks randomly distributed during the whole procedure, and a between-subjects counterbalanced order.

Participants did not receive any explicit information about the different between-blocks contextual uncertainty levels. At the end of the S1-S2 paradigm, the experimenter ensured that the exact S1-S2 probabilistic ratio had remained implicit for each participant by asking if they had caught any relationship between S1 and S2. Participants only reported to have caught a general within-trials emotional congruency or incongruency, but none of them reported neither the precise probabilistic ratio nor to have caught the block manipulation.

Finally, participants completed a self-paced computerized Emotional Recognition Task. For each S1, participants were asked to select which emotion best represented the facial expression displayed, choosing between the following options: anger, disgust, fear, happiness, sadness, surprise, and no emotion. To avoid automaticity in responding, the order of choice options was randomized. The percentage of correct responses was computed for each participant. The Emotional Recognition Task was used to check whether all participants correctly recognized S1 valence. No participant scored lower than 60% accuracy in the task, therefore we could reliably exclude that their predictions could have been affected by a misled S1 valence recognition.

At the end of the experimental session each participant was thanked for the participation and was given detailed information about the research question and objectives of the study.

### Electrophysiological recordings, brain source modelling and data analysis

During the S1-S2 paradigm, the EEG was continuously recorded at a 500 Hz sampling rate through a pre-cabled 128-channel HydroCel Geodesic Sensor Net (HCGSN-128), using a Geodesic high-density EEG System (EGI® GES-300). All electrodes were referenced online to the vertex, and scalp voltages were amplified through a 24-bit DC amplifier (Net Amps™ 300). The impedance was kept below 60 kΩ for each sensor. Signal preprocessing was accomplished offline through the MATLAB toolbox EEGLAB 14.1.2b [48]. The EEG signal was downsampled at 250 Hz and filtered with a digital band-pass filter (0.01–40 Hz, -6dB) using a Hamming windowed sinc finite impulse response filter (filter order = 82500). To compute the ERPs, the continuous EEG was segmented into 4500 ms epochs, from 500 ms before to 4000 ms after S1 onset. The signal was then baseline-corrected to two different baselines: from 500 ms before to the onset of S1 for the purpose of S1-locked ERP analysis; from 200 ms before to the onset of S2 for the purpose of S2-locked ERP analysis. Bad channels were reconstructed with the spherical spline interpolation method [49,50]. Epochs were then digitally inspected through the TBT EEGLAB plug-in, which was applied to electrodes from E40 to E100 (frontal electrode sites were excluded because no eye blinks/movements correction was yet applied). The TBT algorithm performed an automatic rejection of epochs and interpolation of channels on an epoch-by-epoch basis: channels that exceeded a differential average amplitude of ±150 μV were identified and marked for rejection. Channels marked as bad on more than 30% of all epochs were excluded and subsequently interpolated. Epochs having more than 10 bad channels were also excluded. The Infomax algorithm [51] implemented in EEGLAB was used to perform Independent Component Analysis [52]. Artifact-reduced data were decomposed into independent components, which were visually inspected to discard those related to eye blinks, eye movements, heartbeat, and muscular signals, according to their morphology and scalp distribution. The remaining components were then projected back to the electrode space to obtain cleaner EEG epochs. Epochs were further visually inspected and residual artifact-contaminated trials were rejected. Experimental conditions did not differ for final number of epochs accepted (see S1 Table). Data were finally re-referenced to the average of all electrodes. Individual average and grand average ERPs were computed for all experimental conditions, applying a weighted average in order to control for any potential unbalanced number of epochs per condition [53,54].

The ERPs statistical analysis was performed via Brainstorm software, using the Fieldtrip functions [55,56]. A whole-brain paired two-tailed *t*-test (*α* = .05) permutation approach [57,58] was used, performing 1000 Monte-Carlo cluster-based corrected permutations over all 128 channel locations. This approach allows to effectively control the familywise error rate, and it can be used also for complex factorial designs, since providing greater statistical power than traditional spatiotemporal averaging approaches when applied to *a priori* defined time windows [57–59]. The permutations were computed over 6 *a priori* time windows, corresponding to 4 distinguishable ERP components: S1-locked *N170* (140–180 ms) for prediction *generation*; S1-locked early *CNV* (eCNV, 1500–2000 ms) and late *CNV* (lCNV, 2000–2500 ms) for prediction *implementation*; S2-locked *P2* (200–300 ms), early *LPP* (eLPP, 400–600 ms) and late *LPP* (lLPP, 600–800 ms) for prediction *updating*. Interaction effects were tested through a difference-based strategy widely used in the literature, which allowed to further reduce the familywise error rate [59]. All the planned paired-wise comparisons performed are summarized in S1 File. Based on the results of the permutation analysis, ERPs were extracted as the mean voltage amplitude in the abovementioned time windows from the following electrode clusters: an occipital cluster (E70, E74, E75, E81, E82, E83) for the N170, a left-central cluster (E40, E41, E42, E46, E47) for eCNV and lCNV, a parietal cluster (E67, E71, E72, E75, E76, E77) for the P2, and a slightly different parietal cluster (E60, E61, E62, E67, E72, E77, E78, E85) for eLPP and lLPP.

The cortical sources of ERP activity were modeled via Brainstorm software [55]. ICBM152 anatomical template [60] was used to approximate the individual anatomies of each participant and warped to the EEG sensor positions, by employing rigid rotations and translations of digitized landmarks. The conductive head volume was modeled according to the realistic forward model OpenMEEG BEM [61]. The source space was constrained to the cortex and modeled as a grid of 15002 orthogonal current dipole triplets normally oriented to the cortical surface. The inverse modeling was based on sLORETA algorithm and implemented with default parameter settings. This algorithm has proved to be a good source estimator, leading to small localization errors even with low signal-to-noise ratio [62]. The noise covariance matrix was computed from the average of EEG baselines. For each participant, sources were projected to a standard anatomical template (MNI) and their activity was transformed in absolute Z scores relative to the baselines. Finally, a spatial smooth with a FWHM of 3 mm was applied to each source. Cortical activations were located according to the anatomical Destrieux atlas [63] adapted for cortical space solution. Based on each ERP source reconstruction, source map vertices were clustered in the following regions of interest (ROI): right superior temporal sulcus (r-STS) for the N170; left anterior cingulate cortex (l-ACC), left supplementary motor area (l-SMA), and left dorsal-posterior cingulate cortex (l-dPCC) for the eCNV and lCNV; bilateral temporo-parietal junction (TPJ) for the P2; right orbitofrontal cortex (r-OFC), and right temporal pole for the eLPP and lLPP. Absolute values of each ROI were time-averaged from the pertaining ERP time windows, extracted for each participant, and transformed using a natural logarithm.

To assess whether IUS score predicted the neural activity during the task both at the scalp and at the source level, separate linear mixed-effects models (R package: lme4 [64]) with individual random intercept were estimated, with each averaged ERP and ROI activity as dependent variables, *IUS* as a fixed continuous predictor, *S1/S2 valence*, *block*, and the interaction *IUS* × *S1/S2 valence* × *block* as fixed factors. The slopes of the *IUS* trend for each level of the factors (*block*, *S1/S2 valence*) were estimated, and their pairwise differences were tested by means of post-hoc Tukey test (R package: emmeans [65]).

## Results

### ERPs and cortical sources reconstruction

#### Prediction *generation* stage–N170 (140–180 ms) to S1 onset

**Fig 1:**
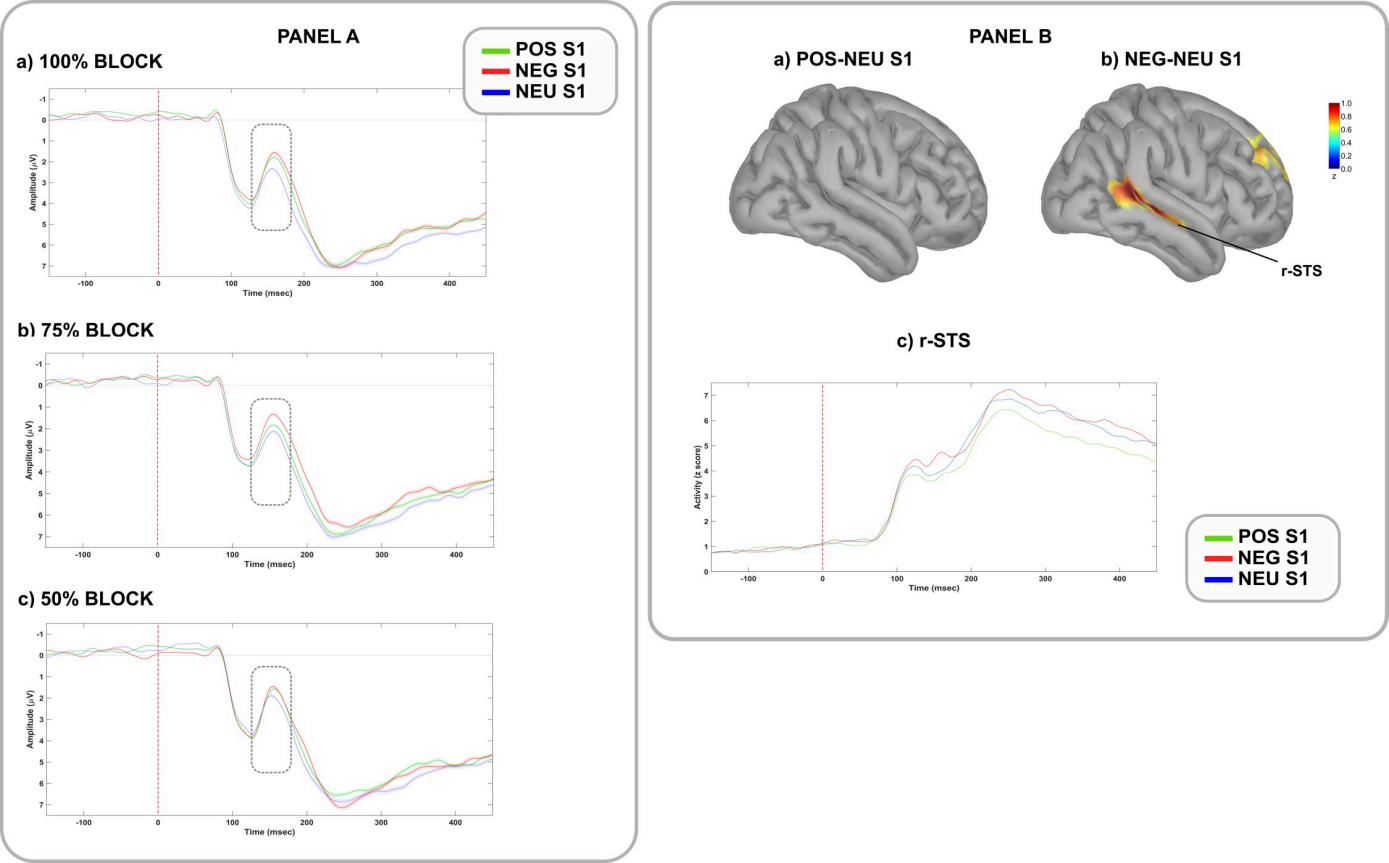
Modulation of ERPs and brain sources during prediction *generation* stage. PANEL A: Grand average ERP waveforms following negative (red lines), positive (green lines) and neutral (blue lines) faces (S1) in the (a) *100%*, (b) *75%* and (c) *50%* blocks. Waveforms are plotted from an occipital cluster of electrodes (E70, E74, E75, E81, E82, E83). Shaded areas denote standard error. The N170 was computed between 140 and 180 msec from S1 onset (time 0). PANEL B: On the top, cortical maps reconstruction of the (a) *POS-NEU* and (b) *NEG-NEU* differences in brain activations in the N170 temporal window (140–180 ms), regardless of blocks. On the bottom, time course of (c) r-STS activations to positive (green lines), negative (red lines), and neutral (blue lines) faces, regardless of blocks. r-STS = right superior temporal sulcus.

A significant negative occipital cluster, reflecting a larger N170, was found in *100%* block when comparing emotional with neutral faces (*POS* vs. *NEU p* = .024, cluster statistic (c) = -476, cluster size (s) = 153; *NEG* vs. *NEU p* = .004, c = -871, s = 246), in *75%* block when comparing fearful with positive and neutral faces (*NEG* vs. *NEU p* = .002, c = -1080, s = 306; *NEG* vs. *POS p* = .018, c = -455, s = 178), and in *50%* block when comparing positive with neutral faces (*POS* vs. *NEU p* = .03, c = -413, s = 164) (Fig 1, panel A). No significant effects were found when comparing difference waves between blocks.

N170 source reconstruction highlighted an emotional modulation on the right superior temporal sulcus (r-STS), showing a maximum pattern of activation after fearful faces (Fig 1, panel B). This pattern replicated consistently across blocks.

#### Prediction *implementation* stage–eCNV (1500–2000 ms) and lCNV (2000–2500 ms) to S1 onset

Permutation analyses showed no significant effects of *block*, nor interaction effects with *S1 valence* on both early and late CNV. Nevertheless, visual inspection of grand average ERP waveforms from a central-left cluster of electrodes showed some amplitude differences, suggesting that a slightly larger CNV could have been elicited in the *50%* block as compared to the others (see S1 Fig). However, since this result is not supported by statistics, it will not be further discussed.

CNV source analysis showed the involvement of a left network, extending over the l-ACC, the supplementary motor area (l-SMA), and the posterior-dorsal part of the cingulate gyrus (l-dPCC).

#### Prediction *updating* stage–P2 (200–300 ms), eLPP (400–600 ms), and lLPP (600–800 ms) to S2 onset

**Fig 2:**
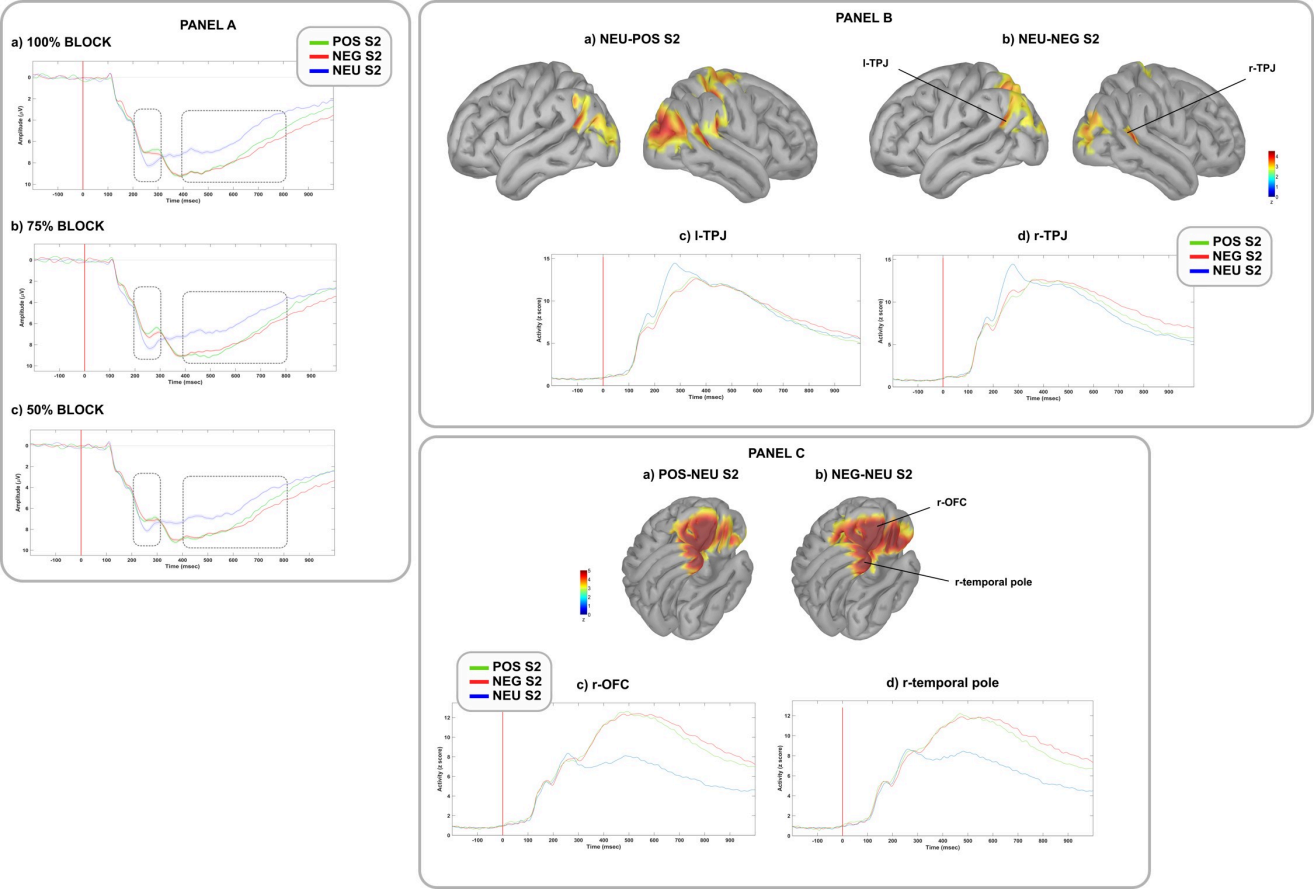
Modulation of ERPs and brain sources during prediction *updating* stage. PANEL A: Grand average ERP waveforms following negative (red lines), positive (green lines), and neutral (blue lines) pictures (S2) in the (a) *100%*, (b) *75%*, and (c) *50%* blocks. Waveforms are plotted from a parietal cluster of electrodes (E60, E61, E62, E67, E71, E72, E75, E76, E77, E78, E85). Shaded areas denote standard error. P2 was computed between 200 and 300 msec, and LPP between 400 and 800 msec from S2 onset (time 0). PANEL B: On the top, cortical maps reconstruction of the (a) *NEU-POS* and (b) *NEU-NEG* differences in brain activations in the P2 temporal window (200–300 ms). On the bottom, time course of (c) l-TPJ, and (d) r-TPJ activations to positive (green lines), negative (red lines), and neutral (blue lines) pictures, regardless of blocks. l-TPJ = left temporoparietal junction, r-TPJ = right temporoparietal junction. PANEL C: On the top, cortical maps reconstruction of the (a) *POS-NEU* and (b) *NEG-NEU* differences in brain activations in the total LPP temporal window (400–800 ms). On the bottom, time course of (c) r- OFC and (d) r-temporal pole activations to positive (green lines), negative (red lines), and neutral (blue lines) pictures, regardless of blocks. r-OFC = right orbitofrontal cortex.

A significant negative parietal cluster was found, signaling a reduced positivity to positive and negative, and so a larger P2 to neutral pictures in all the blocks. Furthermore, a larger P2 was found in *100%* block comparing negative to positive pictures (Table 1; Fig 2, panel A). No significant effects were found when comparing difference waves between blocks.

**Table 1:** Results of planned comparisons in the prediction *updating* stage.

	P2							eLPP							lLPP						
	POS		NEU		*c*	*s*	*p*	POS		NEU		*c*	*s*	*p*	POS		NEU		*c*	*s*	*p*
	*M*	*SD*	*M*	*SD*				*M*	*SD*	*M*	*SD*				*M*	*SD*	*M*	*SD*			
**100%**	7.636	4.024	8.579	3.812	-3075	754	**.002**	8.459	3.890	6.238	3.443	14375	2712	**.002**	6.335	3.232	4.140	2.714	13257	2567	**.002**
**75%**	7.453	4.327	8.732	4.181	-3989	958	**.002**	8.410	4.228	5.979	3.461	15735	2673	**.002**	6.390	3.733	4.308	3.240	12491	2385	**.002**
**50%**	7.635	3.909	8.346	3.814	-2931	842	**.002**	8.154	3.374	6.154	3.458	13251	2711	**.002**	6.022	3.120	4.552	3.122	12987	2525	**.002**
	**NEG**		**NEU**		*c*	*s*	*p*	**NEG**		**NEU**		*c*	*s*	*p*	**NEG**		**NEU**		*c*	*s*	*p*
	*M*	*SD*	*M*	*SD*				*M*	*SD*	*M*	*SD*				*M*	*SD*	*M*	*SD*			
**100%**	7.457	3.910	8.579	3.812	-4745	1088	**.002**	8.568	3.994	6.238	3.443	15813	2782	**.002**	6.867	3.434	4.140	2.714	16155	2770	**.002**
**75%**	7.663	3.929	8.732	4.181	-4642	1036	**.002**	8.336	3.910	5.979	3.461	16376	2928	**.002**	6.722	3.475	4.308	3.240	16235	2930	**.002**
**50%**	7.401	3.669	8.346	3.814	-3285	915	**.002**	8.269	3.685	6.154	3.458	15592	2980	**.002**	6.641	3.279	4.552	3.122	16310	2951	**.002**
	**NEG**		**POS**		*c*	*s*	*p*	**NEG**		**POS**		*c*	*s*	*p*	**NEG**		**POS**		*c*	*s*	*p*
	*M*	*SD*	*M*	*SD*				*M*	*SD*	*M*	*SD*				*M*	*SD*	*M*	*SD*			
**100%**	7.457	3.910	7.636	4.024	-1926	571	**.002**	8.568	3.994	8.459	3.890	670	287	.228	6.867	3.434	6.335	3.232	1701	657	**.04**
**75%**	7.663	3.929	7.453	4.327	-215	85	.382	8.336	3.910	8.410	4.228	391	161	.414	6.722	3.475	6.390	3.733	268	105	.545
**50%**	7.401	3.669	7.635	3.909	-441	176	.158	8.269	3.685	8.154	3.374	774	297	.194	6.641	3.279	6.022	3.120	1132	434	.08

*p*-values (*p*), cluster statistic (*c*), and cluster size (*s*) for each planned comparison (*POS* vs. *NEU*, *NEG* vs. *NEU*, *NEG* vs. *POS*) within each block (*100%*, *75%*, *50%*) in the P2 (200–300 ms), eLPP (400–600 ms) and lLPP (600–800 ms) time windows.

P2 source reconstruction highlighted a larger activation of bilateral temporo-parietal junction (TPJ) to neutral than emotional pictures in all the blocks (Fig 2, panel B).

A significant positive parietal cluster was found, confirming the effect of S2 valence on LPP: positive and negative pictures elicited a larger early and late LPP than neutral pictures in all the blocks. Furthermore, a larger lLPP was found in *100%* block comparing negative with positive pictures (Table 1; Fig 2, panel A). No significant effects were found when comparing difference waves between blocks.

LPP source analysis showed that, as compared with neutral, emotional stimuli elicited a larger activation of the right orbitofrontal cortex (r-OFC) and the right temporal pole in all the blocks (Fig 2, panel C).

### Mixed-effects models

#### Prediction *generation* stage–N170 and r-STS activity (140–180 ms to S1 onset)

No significant main nor interaction effects of the predictors (*IUS*, *S1/S2 valence*, *block*, and their interaction) were found for N170 and r-STS activity (fitted models are summarized in S2 Table).

#### Prediction *implementation* stage–early (1500–2000 ms to S1 onset) and late (2000–2500 ms to S1 onset) CNV, l-ACC, l-SMA, and l-dPCC activity

**Fig 3:**
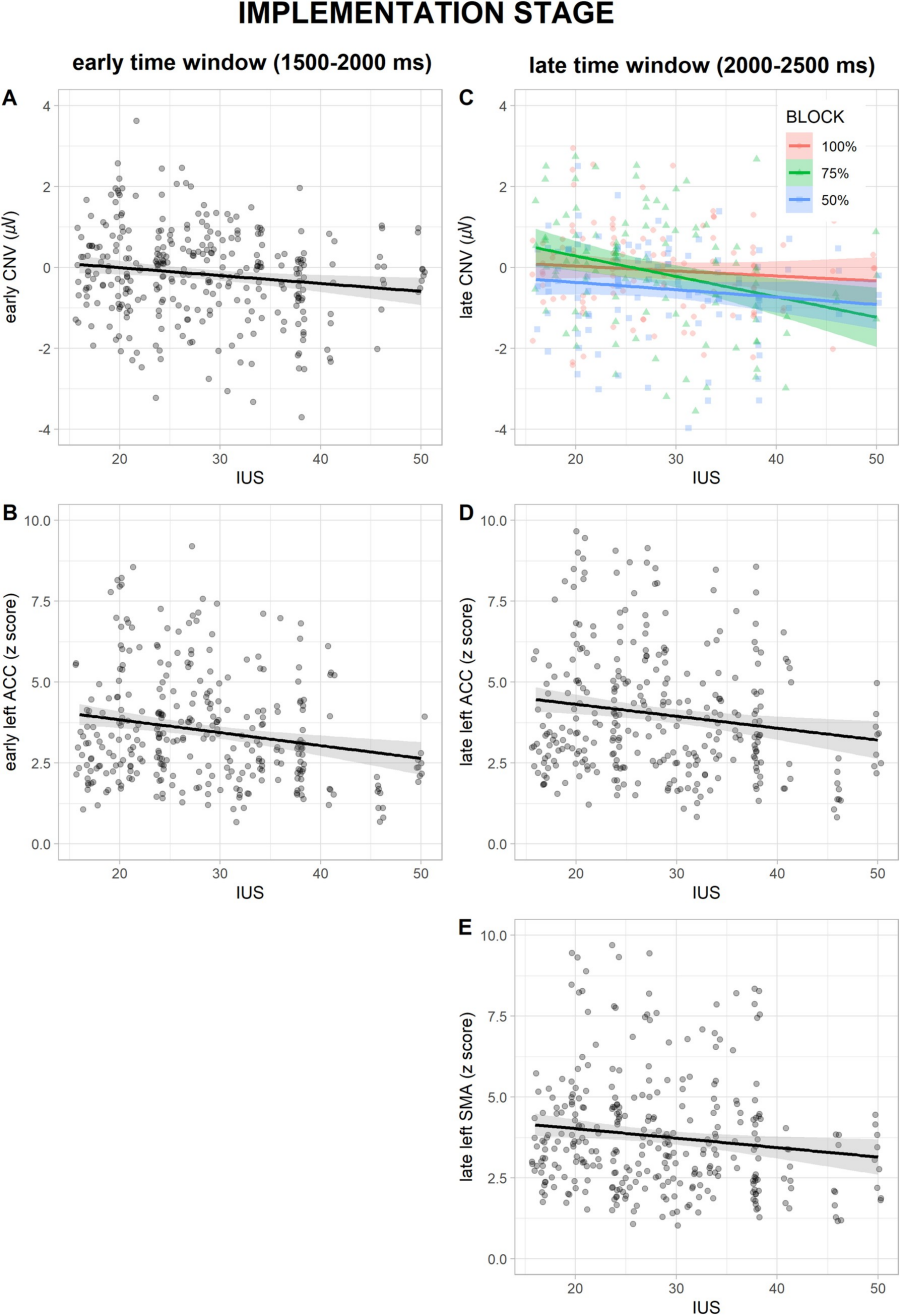
Regression plots in the two time windows of the prediction *implementation* stage. Relationships between IUS scores and (A) early CNV amplitude, (B) early l-ACC activity, (C) late CNV amplitude in *100%* (in red), *75%* (in green), and *50%* (in blue) blocks, (D) late l-ACC and (E) late l-SMA activity. Shaded areas denote the 95% confidence interval.

In the early time window (1500–2000 ms), a significant main effect of *IUS* was found for eCNV amplitude (F_(1, 34)_ = 4.4, *p* = .044, η^2^ = .014, Fig 3A) and early l-ACC activity (F_(1, 34)_ = 6.29, *p* = .017, η^2^ = .021, Fig 3B): higher IUS score predicted larger eCNV negativity and smaller early l-ACC activity regardless of block and S1 valence. No other significant main effects or interactions were found for eCNV, and early l-ACC, l-SMA and l-dPCC activity (fitted models are summarized in Tables 2 and S3).

**Table 2:** Summary of fitted models in the prediction *implementation* stage.

	eCNV			early l-ACC			early l-SMA			early l-dPCC			lCNV			late l-ACC			late l-SMA			late l-dPCC		
*Predictors*	*F*	*DF*	*p*	*F*	*DF*	*p*	*F*	*DF*	*p*	*F*	*DF*	*p*	*F*	*DF*	*p*	*F*	*DF*	*p*	*F*	*DF*	*p*	*F*	*DF*	*p*
block	1.73	2, 272	0.179	1.12	2, 272	0.328	2.38	2, 272	0.094	0.27	2, 272	0.767	4.07	2, 272	**0.018**	1.27	2, 272	0.283	1.89	2, 272	0.153	0.48	2, 272	0.620
valence	0.58	2, 272	0.560	0.02	2, 272	0.976	0.32	2, 272	0.724	0.39	2, 272	0.674	0.54	2, 272	0.584	0.37	2, 272	0.691	0.84	2, 272	0.431	1.40	2, 272	0.249
IUS	4.40	1, 34	**0.044**	6.29	1, 34	**0.017**	3.59	1, 34	0.067	3.84	1, 34	0.058	6.92	1, 34	**0.013**	4.69	1, 34	**0.037**	4.17	1, 34	**0.049**	1.59	1, 34	0.215
block × valence	0.51	4, 272	0.732	0.77	4, 272	0.545	0.74	4, 272	0.563	0.35	4, 272	0.847	0.81	4, 272	0.519	0.57	4, 272	0.686	0.60	4, 272	0.665	1.05	4, 272	0.384
block × IUS	1.68	2, 272	0.189	0.48	2, 272	0.618	1.86	2, 272	0.158	0.45	2, 272	0.641	3.48	2, 272	**0.032**	0.32	2, 272	0.725	1.51	2, 272	0.223	0.33	2, 272	0.716
valence × IUS	0.63	2, 272	0.534	0.07	2, 272	0.929	0.36	2, 272	0.695	0.41	2, 272	0.662	0.75	2, 272	0.475	0.42	2, 272	0.659	0.76	2, 272	0.467	1.33	2, 272	0.265
block × valence × IUS	0.51	4, 272	0.728	0.61	4, 272	0.654	0.56	4, 272	0.691	0.48	4, 272	0.750	0.71	4, 272	0.587	0.48	4, 272	0.749	0.66	4, 272	0.618	1.02	4, 272	0.399

*F*-values (*F*), degrees of freedom (*DF*) and *p*-values (*p*) for each main and interaction effects of predictors on early and late CNV amplitude, early and late left ACC, SMA, and dPCC activity.

As for the late time window (2000–2500 ms), analysis of lCNV showed significant main effects of *IUS* (F_(1, 34)_ = 6.92, *p* = .013, η^2^ = .022) and *block* (F_(2, 272)_ = 4.07, *p* = .018, η^2^ = .025), better explained by a significant *IUS* × *block* interaction (F_(2, 272)_ = 3.48, *p* = .032, η^2^ = .022, Fig 3C): a statistically significant difference in the relationship between IUS and lCNV was found between blocks, regardless of S1 valence. The slope analysis revealed that the slope of the relationship between IUS score and lCNV amplitude was statistically different from 0 only in the *75%* block (*b* = -0.061, *b*_SE_ = 0.017, *95% CI* = -0.0940, -0.0286), suggesting that higher IUS score predicted larger lCNV amplitude only in the *75%* block, regardless of S1 valence. Post-hoc contrasts showed a significant difference between the slopes in the *75%* vs *100%* block (*t*_(272)_ = -2.42, *p* = .043), with more negative values in the first. A significant main effect of *IUS* was found for late l-ACC (F_(1, 34)_ = 4.69, *p* = .037, η^2^ = .016, Fig 3D) and l-SMA activity (F_(1, 34)_ = 4.17, *p* = .049, η^2^ = .014, Fig 3E): higher IUS score predicted smaller late l-ACC and l-SMA activity regardless of block and S1 valence. No other significant main effects or interactions were found for lCNV, and late l-ACC, l-SMA and l-dPCC activity (fitted models are summarized in Tables 2 and S4).

#### Prediction *updating* stage–P2 and bilateral TPJ (200–300 ms to S2 onset), early (400–600 ms to S2 onset) and late (600–800 ms to S2 onset) LPP, r-OFC and r-temporal pole activity

**Fig 4:**
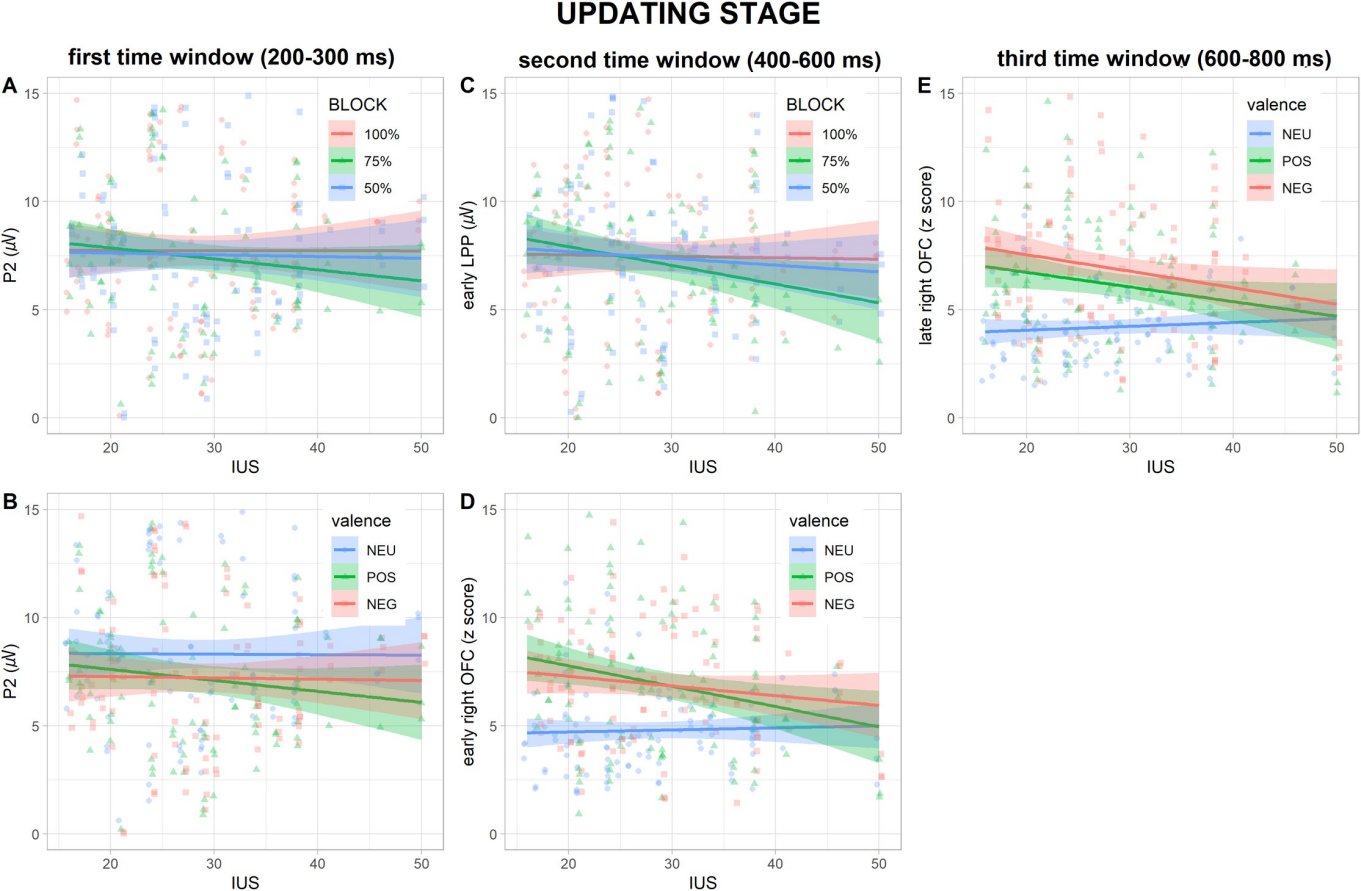
Regression plots in the three time windows of the prediction *updating* stage. Relationships between IUS scores and (A) P2 amplitude in *100%* (in red), *75%* (in green), and *50%* (in blue) blocks, (B) P2 amplitude to *neutral* (in blue), *positive* (in green), and *negative* (in red) S2s, (C) early LPP amplitude in *100%* (in red), *75%* (in green), and *50%* (in blue) blocks, (D) early and (E) late r-OFC activity to *neutral* (in blue), *positive* (in green), and *negative* (in red) S2s. Shaded areas denote the 95% confidence interval.

In the first time window (200–300 ms), analysis of P2 showed a significant main effect of *block* (F_(2, 272)_ = 4.12, *p* = .017, η^2^ = .027), better explained by the significant *IUS* × *block* interaction (F_(2, 272)_ = 3.98, *p* = .02, η^2^ = .026, Fig 4A), and a significant *IUS* × *S2 valence* interaction (F_(2, 272)_ = 4.45, *p* = .013, η^2^ = .029, Fig 4B): a statistically significant difference in the relationship between IUS and P2 amplitude was found between blocks regardless of S2 valence, and between S2 valence levels regardless of block. Nevertheless, the slope analysis revealed that the slopes of the relationship between IUS score and P2 amplitude were not statistically different from 0 in any of the block and S2 valence levels. Post-hoc contrasts showed significant differences between the slopes in the *75%* vs *100%* block (*t*_(272)_ = -2.72, *p* = .019), and between *POS* vs. *NEU* S2 valence levels (*t*_(272)_ = -2.9, *p* = .011), with more negative slope values for the former. Furthermore, a significant main effect of *block* (F_(2, 272)_ = 3.44, *p* = .034, η^2^ = .022) was found for l-TPJ activity. Nevertheless, since overall predictive effects have already been tested by means of permutation analyses, and no effects of block were found on P2 amplitude and its corresponding neural sources, this result will not be further commented. No other significant main effects or interactions were found for P2, and bilateral TPJ activity (fitted models are summarized in Tables 3 and S5).

**Table 3:** Summary of fitted models in the prediction *updating* stage.

	P2			r-TPJ			l-TPJ			eLPP			early r-OFC			early r-temporal pole			lLPP			late r-OFC			late r-temporal pole		
*Predictors*	*F*	*DF*	*p*	*F*	*DF*	*p*	*F*	*DF*	*p*	*F*	*DF*	*p*	*F*	*DF*	*p*	*F*	*DF*	*p*	*F*	*DF*	*p*	*F*	*DF*	*p*	*F*	*DF*	*p*
block	4.12	2, 272	**0.017**	2.53	2, 272	0.082	3.44	2, 272	**0.034**	3.82	2, 272	**0.023**	1.18	2, 272	0.308	0.94	2, 272	0.392	1.28	2, 272	0.281	0.97	2, 272	0.382	0.83	2, 272	0.438
valence	2.15	2, 272	0.119	0.92	2, 272	0.401	0.83	2, 272	0.436	17.15	2, 272	**< 0.001**	23.15	2, 272	**< 0.001**	4.74	2, 272	**0.01**	13.11	2, 272	**< 0.001**	19.12	2, 272	**< 0.001**	9.47	2, 272	**< 0.001**
IUS	0.17	1, 34	0.68	0.06	1, 34	0.805	0.07	1, 34	0.787	0.29	1, 34	0.596	1.13	1, 34	0.296	1.2	1, 34	0.28	0.11	1, 34	0.748	0.96	1, 34	0.335	1.57	1, 34	0.219
block × valence	0.97	4, 272	0.423	0.27	4, 272	0.897	1.76	4, 272	0.138	0.53	4, 272	0.715	0.75	4, 272	0.56	1.85	4, 272	0.119	0.41	4, 272	0.798	0.84	4, 272	0.503	2.16	4, 272	0.073
block × IUS	3.98	2, 272	**0.02**	2.11	2, 272	0.124	2.33	2, 272	0.099	4.69	2, 272	**0.01**	1.01	2, 272	0.367	0.51	2, 272	0.603	1.26	2, 272	0.285	0.87	2, 272	0.418	0.47	2, 272	0.625
valence × IUS	4.45	2, 272	**0.013**	1.68	2, 272	0.188	2.22	2, 272	0.111	2.06	2, 272	0.13	8.77	2, 272	**< 0.001**	0.65	2, 272	0.523	1.94	2, 272	0.145	6.45	2, 272	**0.002**	2.75	2, 272	0.066
block × valence × IUS	1.01	4, 272	0.405	0.63	4, 272	0.64	1.87	4, 272	0.116	0.78	4, 272	0.537	0.5	4, 272	0.738	1.55	4, 272	0.189	0.58	4, 272	0.681	0.62	4, 272	0.649	1.94	4, 272	0.103

*F*-values (*F*), degrees of freedom (*DF*) and *p*-values (*p*) for each main and interaction effects of predictors on P2, early and late LPP amplitude, bilateral TPJ, early and late right OFC and temporal pole activity.

In the second time window (400–600 ms), analysis of the eLPP showed a significant main effect of *block* (F_(2, 272)_ = 3.82, *p* = .023, η^2^ = .023), better explained by the significant *IUS* × *block* interaction (F_(2, 272)_ = 4.69, *p* = .01, η^2^ = .028, Fig 4C), and a main effect of *S2 valence* (F_(2, 272)_ = 17.15, *p* < .001, η^2^ = .103): a statistically significant difference in the relationship between IUS and eLPP amplitude was found between blocks regardless of S2 valence, while the main effect of S2 valence replicates the results of the permutation analysis suggesting a larger eLPP for emotional as compared to neutral S2s. Nevertheless, the slope analysis revealed that the slopes of the relationship between IUS score and eLPP amplitude were not statistically different from 0 in any of the blocks. Post-hoc contrasts showed significant differences between the slopes in the *75%* vs *100%* block (*t*_(272)_ = -2.92, *p* = .011), with more negative slope values for the former. Furthermore, a significant main effect of *S2 valence* (F_(2, 272)_ = 23.15, *p* < .001, η^2^ = .133), better explained by the significant *IUS* × *S2 valence* interaction (F_(2, 272)_ = 8.77, *p* < .001, η^2^ = .05, Fig 4D) was found for the early r-OFC activity: a statistically significant difference in the relationship between IUS and early r-OFC activity was found between S2 valence levels regardless of blocks. Nevertheless, the slope analysis revealed that the slopes of the relationship between IUS score and early r-OFC activity were not statistically different from 0 in any of the S2 valence levels. Post-hoc contrasts showed significant differences between the slopes in the *POS* vs. *NEU* and *NEG* vs. *NEU* S2 valence levels (*t*_(272)_ = -3.89 and -3.29, *p* < .011 and = .003, respectively), with more negative slope values for the emotional as compared to the neutral S2 valence levels. Lastly, a significant main effect of *S2 valence* (F_(2, 272)_ = 4.74, *p* = .01, η^2^ = .031) was found for r-temporal pole activity, replicating the larger activation to emotional stimuli already shown by LPP source reconstruction. No other significant main effects or interactions were found for eLPP, and early r-OFC and r-temporal pole activity (fitted models are summarized in Tables 3 and S6).

In the third time window (600–800 ms), analysis of the late r-OFC activity showed a significant main effect of *S2 valence* (F_(2, 272)_ = 19.12, *p* < .001, η^2^ = .113), better explained by a significant *IUS* × *S2 valence* interaction (F_(2, 272)_ = 6.45, *p* = .002, η^2^ = .038, Fig 4E): a statistically significant difference in the relationship between IUS and late r-OFC activity was found between S2 valence levels regardless of blocks. Nevertheless, the slope analysis revealed that the slopes of the relationship between IUS score and late r-OFC activity were not statistically different from 0 in any of the S2 valence levels. Post-hoc contrasts showed significant differences between the slopes in the *POS* vs. *NEU* and *NEG* vs. *NEU* S2 valence levels (*t*_(272)_ = -3.14 and -3.08, *p* = .005 and .006, respectively), with more negative slope values for the emotional as compared to the neutral S2 valence levels. A significant main effect of *S2 valence* was found for lLPP (F_(2, 272)_ = 13.11, *p* < .001, η^2^ = .084) and r-temporal pole activity (F_(2, 272)_ = 9.47, *p* < .001, η^2^ = .059), replicating results already shown by permutation analyses and corresponding source reconstruction. No other significant main effects or interactions were found for lLPP, and late r-OFC and r-temporal pole activity (fitted models are summarized in Tables 3 and S7).

## Discussion

To our knowledge, this study represents the first attempt to investigate the modulating role of IU on the neural activity elicited during the *generation*, *implementation*, and *updating* stages of emotional predictions, as a function of contexts with fully valid (*100%*), moderately valid (*75%*), or random (*50%*) predictive values.

Independent of IU, both contextual uncertainty and emotional valence exerted a detectable influence on the neural correlates of emotional predictions, especially during the *generation* and *updating* stages. Though a detailed discussion of these effects goes beyond the aims of this study, a brief summary of the main findings is still needed. During prediction *generation*, S1 valence modulated the N170 towards a processing advantage for emotional faces, with slight valence-specific differences among predictive contexts. This effect was subtended by a higher cortical activity in the r-STS, an area involved in the perceptual coding of changeable facial properties, among which emotional facial expression [66]. The N170/r-STS modulation is coherent with the extraction of emotional and contextual information from the environment, involving domain-specific brain circuits, and assumed to be crucial to continuously generate predictions [15,16]. During prediction *updating*, a strong emotional modulation emerged on the P2 and LPP components across all blocks: larger LPPs were found for emotional than neutral pictures, whereas the opposite was observed for P2s. The latter effect could index the infrequency of neutral S2s, and the consequent updating of the overall predictive model, subtended by a coherent modulation in the TPJ, an area involved in contextual updating [37,67]. The LPP modulation replicated a well-established effect in the literature, signaling a greater motivated attention towards emotional stimuli, subtended by domain-specific neural circuits (i.e., r-OFC, r-temporal pole) [38,39].

Concerning the main aim of this study, that is investigating the modulating role of IU on the neural activity elicited during the *generation*, *implementation*, and *updating* stages, the following results emerged. Contrary to what hypothesized, but in line with some previous evidence [19,22], individual differences in IU did not affect the prediction *generation* stage, either at the ERP or at the source level, suggesting that the extraction of emotional and contextual information conveyed by the faces develops in a similar manner along the different IU levels.

During the *implementation* stage, IU exerted a modulating role both on ERPs and sources activity, in partial consistency with our hypotheses, and with interesting differences as a function of time. In the early *implementation* stage, a higher IU was associated with a context- and valence-independent decrease of neural activity within l-ACC, an area assumed to subtend the behavioral and cognitive avoidance of exposure to uncertainty [6], and the continuous assessment of environmental uncertainty levels [68]. Net of the needed caution in interpreting brain sources activity as reconstructed from EEG, a reduced l-ACC activation to increasing IU levels could signal a disrupted environmental assessment of uncertainty. This could in turn translate into a heightened deployment of anticipatory resources, as reflected in larger eCNV amplitudes, in order to try to solve uncertainty [69]. In the late *implementation* stage, instead, higher IU levels were found to predict a context- and valence-independent decrease of neural activity within both l-ACC and l-SMA. So, as the implementation stage proceeds, the disrupted environmental uncertainty assessment, subtended by l-ACC decreased activity, seems to match an inhibition in action plans programming, as suggested by the reduced l-SMA activity, thus potentially contributing to the behavioral inhibition typical of high-IU individuals [4] (cfr. [2,3] for potential relationships with BIS activation). Furthermore, the heightened deployment of anticipatory resources, subtended by a larger CNV and reflecting an attempt to solve uncertainty, specialized into a context-specific effect: IU scores were found to negatively predict larger lCNVs only within the moderately predictive (*75%*) as compared to the fully predictive (*100%*) context. It can be suggested that, as IU increases more anticipatory resources are pre-allocated only in those contexts in which the amount of uncertainty is perceived as “solvable” (i.e., moderately predictive *75%*), while in more certain contexts (i.e., fully predictive *100%*) it could be metabolically effective to save resources, since there is no uncertainty to solve. This interpretation is consistent with IU models suggesting that neural, cognitive and behavioral peculiarities of high-IU individuals are aimed to increase perceived certainty and to reduce uncertainty exposure [4,10].

Within prediction *updating*, results were partially consistent with our hypotheses: higher IU levels were found to be associated both with valence-specific effects, such as smaller P2s to positive S2s and smaller r-OFC activity to emotional S2s, and with context-specific effects, such as smaller P2s and eLPPs to S2s in the moderately predictive (*75%*) context. Although slope analyses failed to find significant differences, and so these results should be considered with caution, some explanations could still be suggested. The valence-specific decrease of P2 amplitude to positive as compared to neutral S2s, reflecting a reduced automatic attention allocation, could represent an early neural correlate of the deficient safety learning which characterizes high-IU individuals [6]. This, in turn, could prevent emotional predictive models from being efficiently updated, by integrating environmental safety cues, because of the reduced attention allocation towards them. Furthermore, higher IU was found to be associated with a context-specific decrease in P2s and eLPPs when comparing the moderately predictive (*75%*) with the fully predictive (*100%*) contexts. This result, suggesting a dampening in S2 processing both at early and later stages, could reflect the uncertainty avoidance and a disrupted prediction error signaling typical of high-IU individuals [6]. Moreover, this result specifically arises in a context in which the updating of emotional predictive models is required (i.e., moderately predictive *75%*), as compared to a context in which there is no need to update internal models since no expectancy violation occurs (i.e., fully predictive *100%*). Lastly, both in the early and late stages of prediction *updating*, IU was found to be associated with a smaller r-OFC activity to emotional as compared to neutral S2s. Since OFC is involved in emotion regulation [70,71], its reduced activity could subtend a limited access to emotion regulation strategies, which is assumed to be a peculiar facet of high-IU traits [72], and which seems to spread across positive and negative valence and also across all predictive contexts.

Overall, our results suggest that IU differently modulated the neural activity developing across the three stages of emotional predictions construction. Prediction *generation* was not affected by individual differences in IU. During the *implementation* stage, instead, higher IU was associated with a disrupted environmental uncertainty assessment, and a consequent inhibition in anticipatory action plans programming, associated with a facilitation in the deployment of computational resources. These processes serve to solve uncertainty, specifically arising in moderately predictive contexts in which uncertainty is perceived as solvable. Finally, IU modulated the *updating* stage by predicting context-dependent uncertainty avoidance, and context-independent reduced attention to safety cues and disrupted access to emotion regulation strategies.

Implications of these results are promising. They demonstrate that individual differences in IU can actually affect how emotional predictions are constructed at a neural level. Given the conceptual relationships between IU, emotional traits and disorders (e.g., neuroticism, anxiety), and the activation of the associated behavioral patterns (e.g., BIS, fight-flight-freeze response), and being IU considered as the lower-order construct accounting for them [2,3], it can be hypothesized that such emotional traits might share similar neural correlates of the construction of emotional predictions. Thus, our results encourage broader considerations, useful for preventive and clinical applications within the domain of affective psychopathology. The CNV component and its neural sources, as well as the P2, the LPP and the r-OFC activity could in fact be considered as potential neural indicators to assess, or to intervene on (e.g., through biofeedback), when developing preventive and treatment measures for clinical and subclinical populations. Furthermore, our results suggest that, when assessing the relationships between IU and emotion, contextual uncertainty should be taken into more careful account, since its amount has proved to impact neural correlates of emotional predictions [26].

In conclusion, some limitations of our study must be addressed. First, the choice to screen out participants showing extreme levels of blood-injection-injury fear might have created a restricted range recruitment bias, and the sample was slightly sex-unbalanced (16 M vs. 20 F). Moreover, the final sample size was relatively small (N = 36), and it allowed to reach a power of about 0.5, lower than the desirable 0.8 level. Nevertheless, this issue was partially counterbalanced by the large number of trials we employed in our experimental paradigm, which enabled to heighten power by increasing the signal-to-noise ratio [73]. Second, a passive viewing task might have contributed to an overall decrease of ERP amplitudes, thus preventing some weaker modulations to reach statistical significance [74]. Furthermore, the lack of behavioral data makes it difficult to draw inferences on how the effects of IU on neural correlates of emotional predictions could translate into behavior and/or subjective affective experience. Lastly, the limited EEG spatial resolution implies caution when interpreting brain sources activations. We encourage future studies to overcome limitations by employing an active task and collecting subjective valence and expectancy ratings.

Net of these limitations, our paradigm still brought several elements of novelty to the literature. First, we used a totally uninstructed experimental task, in which participants were left free to implicitly construct their own predictive models as it occurs in everyday life. Second, IU was modeled as a continuous predictor, thus better approximating the construct, and preventing some slight individual differences to be lost in a dichotomization of the variable. Third, an intermediate level of contextual uncertainty (*75%*) was introduced to better resemble a more realistic, moderately certain environmental context. Fourth, highly arousing emotional stimuli were employed as S1s and S2s, including pleasant stimuli, thus allowing to isolate valence-specific effects. Fifth and last, high-density EEG allowed to investigate the neural correlates of emotional predictions both at the scalp and, with due caution, at the source level, thus conveying a comprehensive neurocomputational evidence.

## Supporting information

S1 Fig Modulation of ERPs and brain sources during prediction *implementation* stage. Grand average ERP waveforms during prediction implementation in the 100% (continuous black line), 75% (continuous grey line), and 50% (dashed black line) blocks. Waveforms are plotted from a central cluster of electrodes (E40, E41, E42, E46, E47). Shaded areas denote standard error. CNV was computed between 1500 and 2500 msec from S1 onset (time 0). For visualization purposes, waveforms were low-pass re-filtered at 10 Hz.
(TIF)

S1 Table Descriptive statistics and ANOVA results on the final number of epochs accepted for each experimental condition. Mean (*M*), standard deviation (*SD*) and *F*-test (*F*) of final epochs number for each level of the independent variables (*block*, *S1/S2 valence*).
(DOCX)

S2 Table Summary of the model fitted on the prediction *generation* stage. Dependent variables: N170 and r-STS.
(DOCX)

S3 Table Summary of the model fitted on the early time window of the prediction *implementation* stage. Dependent variables: eCNV and early l-ACC, l-SMA and l-dPCC.
(DOCX)

S4 Table Summary of the model fitted on the late time window of the prediction *implementation* stage. Dependent variables: lCNV and late l-ACC, l-SMA and l-dPCC.
(DOCX)

S5 Table Summary of the model fitted on the first time window of the prediction *updating* stage. Dependent variables: P2 and bilateral TPJ.
(DOCX)

S6 Table Summary of the model fitted on the second time window of the prediction *updating* stage. Dependent variables: eLPP and early r-OFC and r-temporal pole.
(DOCX)

S7 Table Summary of the model fitted on the third time window of the prediction *updating* stage. Dependent variables: lLPP and late r-OFC and r-temporal pole.
(DOCX)

S1 File Supplementary stimulus material and data analysis information. Stimulus material: NimStim and IAPS picture numbers employed in the experimental paradigm. Data analysis: Planned planned paired-wise comparisons performed in the ERPs permutation analysis.
(PDF)
