# Supplementary figures and images for: Dealing with uncertainty: A high-density EEG investigation on how intolerance of uncertainty affects emotional predictions
Source: PLoS One. 2021 Jul 1;16(7):e0254045. doi: 10.1371/journal.pone.0254045 (PMC8248604; doi:10.1371/journal.pone.0254045)

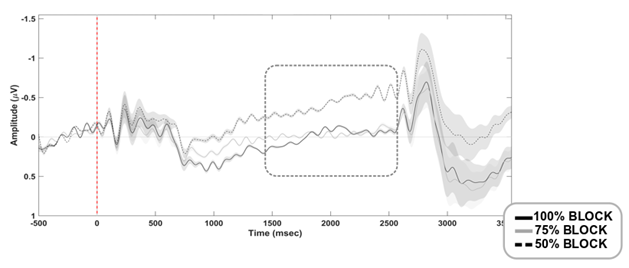

Supplement: S1 Fig — Grand average ERP waveforms during prediction implementation in the 100% (continuous black line), 75% (continuous grey line), and 50% (dashed black line) blocks. Waveforms are plotted from a central cluster of electrodes (E40, E41, E42, E46, E47). Shaded areas denote standard error. CNV was computed between 1500 and 2500 msec from S1 onset (time 0). For visualization purposes, waveforms were low-pass re-filtered at 10 Hz. (TIF) [file pone.0254045.s001.tif]
