# Supplementary material for: Dealing with uncertainty: A high-density EEG investigation on how intolerance of uncertainty affects emotional predictions
Source: PLoS One. 2021 Jul 1;16(7):e0254045. doi: 10.1371/journal.pone.0254045 (PMC8248604; doi:10.1371/journal.pone.0254045)
Supplement: S1 Table — Mean (M), standard deviation (SD) and F-test (F) of final epochs number for each level of the independent variables (block, S1/S2 valence). (DOCX) [file pone.0254045.s002.docx]

|  | **100%** | | **75%** | | **50%** | | *F* (2,105) | *p* |
| --- | --- | --- | --- | --- | --- | --- | --- | --- |
| **Block** | *M* | *SD* | *M* | *SD* | *M* | *SD* | 0.417 | .66 |
|  | 113.33 | 5.87 | 114.56 | 6.39 | 114.17 | 5.07 |  |  |
|  | **POS** | | **NEG** | | **NEU** | |  |  |
| **S1 valence** | *M* | *SD* | *M* | *SD* | *M* | *SD* | 0.516 | .598 |
|  | 113.64 | 4.87 | 114.67 | 4.34 | 113.75 | 4.90 |  |  |
| **S2 valence** | *M* | *SD* | *M* | *SD* | *M* | *SD* | 0.256 | .775 |
|  | 113.72 | 4.93 | 114.47 | 4.62 | 113.86 | 4.64 |  |  |
