# Supplementary material for: Dealing with uncertainty: A high-density EEG investigation on how intolerance of uncertainty affects emotional predictions
Source: PLoS One. 2021 Jul 1;16(7):e0254045. doi: 10.1371/journal.pone.0254045 (PMC8248604; doi:10.1371/journal.pone.0254045)
Supplement: S2 Table — Dependent variables: N170 and r-STS. (DOCX) [file pone.0254045.s003.docx]

|  | **N170** | | | | **r-STS** | | | |
| --- | --- | --- | --- | --- | --- | --- | --- | --- |
| *Predictors* | *Estimates* | *std. Error* | *CI* | *p* | *Estimates* | *std. Error* | *CI* | *p* |
| (Intercept) | 2.53 | 1.58 | -0.66 – 5.73 | 0.118 | 0.95 | 0.28 | 0.39 – 1.50 | **0.001** |
| block50 | 0.28 | 0.81 | -1.31 – 1.88 | 0.725 | -0.18 | 0.26 | -0.69 – 0.34 | 0.499 |
| block50 × IUS | -0.02 | 0.03 | -0.08 – 0.03 | 0.375 | 0.01 | 0.01 | -0.01 – 0.03 | 0.344 |
| block50 × valenceneg | 0.50 | 1.14 | -1.75 – 2.75 | 0.660 | 0.47 | 0.37 | -0.26 – 1.19 | 0.209 |
| block50 × valenceneg × IUS | -0.00 | 0.04 | -0.08 – 0.07 | 0.931 | -0.02 | 0.01 | -0.04 – 0.01 | 0.153 |
| block50 × valencepos | -0.10 | 1.14 | -2.35 – 2.16 | 0.934 | 0.13 | 0.37 | -0.60 – 0.86 | 0.731 |
| block50 × valencepos × IUS | 0.02 | 0.04 | -0.06 – 0.09 | 0.694 | -0.00 | 0.01 | -0.03 – 0.02 | 0.717 |
| block75 | -0.40 | 0.81 | -1.99 – 1.20 | 0.626 | -0.34 | 0.26 | -0.85 – 0.18 | 0.201 |
| block75 × IUS | 0.01 | 0.03 | -0.05 – 0.06 | 0.847 | 0.01 | 0.01 | -0.00 – 0.03 | 0.109 |
| block75 × valenceneg | 0.07 | 1.14 | -2.18 – 2.32 | 0.953 | 0.70 | 0.37 | -0.03 – 1.43 | 0.059 |
| block75 × valenceneg × IUS | -0.01 | 0.04 | -0.08 – 0.07 | 0.860 | -0.03 | 0.01 | -0.05 – -0.00 | **0.034** |
| block75 × valencepos | 0.84 | 1.14 | -1.41 – 3.09 | 0.463 | 0.24 | 0.37 | -0.49 – 0.96 | 0.524 |
| block75 × valencepos × IUS | -0.02 | 0.04 | -0.09 – 0.06 | 0.687 | -0.01 | 0.01 | -0.03 – 0.01 | 0.434 |
| IUS | 0.01 | 0.05 | -0.10 – 0.12 | 0.826 | -0.00 | 0.01 | -0.02 – 0.02 | 0.695 |
| neu | *Reference* |  |  |  | *Reference* |  |  |  |
| valenceneg × IUS | 0.01 | 0.03 | -0.04 – 0.07 | 0.614 | 0.01 | 0.01 | -0.01 – 0.03 | 0.164 |
| pos | -0.99 | 0.81 | -2.58 – 0.60 | 0.222 | -0.02 | 0.26 | -0.54 – 0.49 | 0.924 |
| neg | -1.13 | 0.81 | -2.73 – 0.46 | 0.162 | -0.30 | 0.26 | -0.81 – 0.22 | 0.253 |
| valencepos × IUS | 0.02 | 0.03 | -0.04 – 0.07 | 0.549 | 0.00 | 0.01 | -0.02 – 0.02 | 0.953 |
| **Random Effects** | | | | | | | | |
| σ^2^ | 0.96 | | | | 0.10 | | | |
| τ_00_ | 6.39 _ID_ | | | | 0.13 _ID_ | | | |
| ICC | 0.87 | | | | 0.56 | | | |
| N | 36 _ID_ | | | | 36 _ID_ | | | |
| Observations | 324 | | | | 324 | | | |
| Marginal R^2^ / Conditional R^2^ | 0.016 / 0.872 | | | | 0.012 / 0.566 | | | |

|  | **N170** | | | **r-STS** | | |
| --- | --- | --- | --- | --- | --- | --- |
| *Predictors* | *F* | *DF* | *p* | *F* | *DF* | *p* |
| block | 0.68 | 2, 272 | 0.505 | 0.04 | 2, 272 | 0.959 |
| valence | 2.27 | 2, 272 | 0.106 | 0.25 | 2, 272 | 0.778 |
| IUS | 0.08 | 1, 34 | 0.777 | 0.05 | 1, 34 | 0.832 |
| block × valence | 0.41 | 4, 272 | 0.798 | 0.97 | 4, 272 | 0.425 |
| block × IUS | 1.00 | 2, 272 | 0.369 | 0.08 | 2, 272 | 0.919 |
| valence × IUS | 0.55 | 2, 272 | 0.580 | 0.35 | 2, 272 | 0.704 |
| block × valence × IUS | 0.20 | 4, 272 | 0.940 | 1.23 | 4, 272 | 0.300 |
