# Supplementary material for: Dealing with uncertainty: A high-density EEG investigation on how intolerance of uncertainty affects emotional predictions
Source: PLoS One. 2021 Jul 1;16(7):e0254045. doi: 10.1371/journal.pone.0254045 (PMC8248604; doi:10.1371/journal.pone.0254045)
Supplement: S3 Table — Dependent variables: eCNV and early l-ACC, l-SMA and l-dPCC. (DOCX) [file pone.0254045.s004.docx]

|  | **eCNV** | | | | **early l-ACC** | | | | **early l-SMA** | | | | **early l-dPCC** | | | |
| --- | --- | --- | --- | --- | --- | --- | --- | --- | --- | --- | --- | --- | --- | --- | --- | --- |
| *Predictors* | *Estimates* | *std. Error* | *CI* | *p* | *Estimates* | *std. Error* | *CI* | *p* | *Estimates* | *std. Error* | *CI* | *p* | *Estimates* | *std. Error* | *CI* | *p* |
| (Intercept) | 0.19 | 0.69 | -1.18 – 1.56 | 0.787 | 1.58 | 0.30 | 0.99 – 2.17 | **<0.001** | 1.27 | 0.27 | 0.73 – 1.81 | **<0.001** | 1.35 | 0.29 | 0.78 – 1.92 | **<0.001** |
| block50 | 0.18 | 0.94 | -1.67 – 2.02 | 0.850 | 0.05 | 0.36 | -0.67 – 0.76 | 0.900 | 0.42 | 0.35 | -0.27 – 1.10 | 0.230 | -0.22 | 0.35 | -0.91 – 0.46 | 0.521 |
| block50 × IUS | -0.01 | 0.03 | -0.07 – 0.05 | 0.764 | 0.00 | 0.01 | -0.02 – 0.03 | 0.915 | -0.01 | 0.01 | -0.03 – 0.01 | 0.463 | 0.01 | 0.01 | -0.01 – 0.03 | 0.396 |
| block50 × valenceneg | -0.81 | 1.33 | -3.42 – 1.80 | 0.540 | 0.52 | 0.51 | -0.50 – 1.53 | 0.316 | -0.20 | 0.49 | -1.17 – 0.77 | 0.686 | 0.21 | 0.49 | -0.77 – 1.18 | 0.677 |
| block50 × valenceneg × IUS | 0.01 | 0.05 | -0.08 – 0.10 | 0.771 | -0.02 | 0.02 | -0.05 – 0.02 | 0.287 | -0.00 | 0.02 | -0.03 – 0.03 | 0.992 | -0.01 | 0.02 | -0.04 – 0.02 | 0.600 |
| block50 × valencepos | -0.72 | 1.33 | -3.33 – 1.89 | 0.588 | 0.22 | 0.51 | -0.79 – 1.23 | 0.666 | 0.23 | 0.49 | -0.74 – 1.20 | 0.639 | 0.14 | 0.49 | -0.83 – 1.11 | 0.773 |
| block50 × valencepos × IUS | 0.01 | 0.05 | -0.07 – 0.10 | 0.753 | -0.00 | 0.02 | -0.04 – 0.03 | 0.804 | -0.01 | 0.02 | -0.04 – 0.02 | 0.503 | -0.01 | 0.02 | -0.04 – 0.03 | 0.721 |
| block75 | 1.65 | 0.94 | -0.20 – 3.49 | 0.080 | 0.03 | 0.36 | -0.69 – 0.75 | 0.934 | -0.08 | 0.35 | -0.76 – 0.61 | 0.826 | 0.16 | 0.35 | -0.53 – 0.85 | 0.650 |
| block75 × IUS | -0.06 | 0.03 | -0.12 – 0.00 | 0.053 | -0.00 | 0.01 | -0.03 – 0.02 | 0.746 | 0.00 | 0.01 | -0.02 – 0.03 | 0.834 | -0.00 | 0.01 | -0.03 – 0.02 | 0.687 |
| block75 × valenceneg | -1.85 | 1.33 | -4.46 – 0.77 | 0.165 | 0.09 | 0.51 | -0.92 – 1.10 | 0.865 | 0.45 | 0.49 | -0.52 – 1.42 | 0.363 | -0.00 | 0.49 | -0.98 – 0.97 | 0.995 |
| block75 × valenceneg × IUS | 0.06 | 0.05 | -0.03 – 0.15 | 0.180 | 0.00 | 0.02 | -0.03 – 0.04 | 0.912 | -0.01 | 0.02 | -0.05 – 0.02 | 0.416 | -0.00 | 0.02 | -0.04 – 0.03 | 0.896 |
| block75 × valencepos | -1.12 | 1.33 | -3.73 – 1.49 | 0.398 | 0.56 | 0.51 | -0.45 – 1.57 | 0.277 | 0.66 | 0.49 | -0.30 – 1.63 | 0.178 | -0.38 | 0.49 | -1.35 – 0.59 | 0.446 |
| block75 × valencepos × IUS | 0.04 | 0.05 | -0.05 – 0.13 | 0.415 | -0.01 | 0.02 | -0.04 – 0.03 | 0.683 | -0.02 | 0.02 | -0.06 – 0.01 | 0.162 | 0.01 | 0.02 | -0.02 – 0.05 | 0.402 |
| IUS | -0.01 | 0.02 | -0.06 – 0.04 | 0.708 | -0.01 | 0.01 | -0.03 – 0.01 | 0.148 | -0.01 | 0.01 | -0.03 – 0.01 | 0.448 | -0.02 | 0.01 | -0.04 – 0.00 | 0.106 |
| neu | *Reference* |  |  |  | *Reference* |  |  |  | *Reference* |  |  |  | *Reference* |  |  |  |
| valenceneg × IUS | -0.01 | 0.03 | -0.07 – 0.05 | 0.711 | 0.01 | 0.01 | -0.02 – 0.03 | 0.518 | 0.01 | 0.01 | -0.02 – 0.03 | 0.539 | 0.01 | 0.01 | -0.01 – 0.03 | 0.410 |
| pos | 0.12 | 0.94 | -1.73 – 1.96 | 0.900 | -0.28 | 0.36 | -1.00 – 0.43 | 0.438 | -0.23 | 0.35 | -0.92 – 0.45 | 0.504 | 0.03 | 0.35 | -0.66 – 0.72 | 0.934 |
| neg | 0.37 | 0.94 | -1.48 – 2.22 | 0.692 | -0.25 | 0.36 | -0.96 – 0.47 | 0.496 | -0.18 | 0.35 | -0.86 – 0.51 | 0.610 | -0.24 | 0.35 | -0.93 – 0.45 | 0.490 |
| valencepos × IUS | 0.00 | 0.03 | -0.06 – 0.07 | 0.915 | 0.00 | 0.01 | -0.02 – 0.03 | 0.749 | 0.01 | 0.01 | -0.01 – 0.03 | 0.478 | -0.00 | 0.01 | -0.02 – 0.02 | 0.956 |
| **Random Effects** | | | | | | | | | | | | | | | | |
| σ^2^ | 1.29 | | | | 0.19 | | | | 0.18 | | | | 0.18 | | | |
| τ_00_ | 0.13 _ID_ | | | | 0.07 _ID_ | | | | 0.04 _ID_ | | | | 0.07 _ID_ | | | |
| ICC | 0.09 | | | | 0.25 | | | | 0.19 | | | | 0.28 | | | |
| N | 36 _ID_ | | | | 36 _ID_ | | | | 36 _ID_ | | | | 36 _ID_ | | | |
| Observations | 324 | | | | 324 | | | | 324 | | | | 324 | | | |
| Marginal R^2^ / Conditional R^2^ | 0.063 / 0.147 | | | | 0.085 / 0.318 | | | | 0.057 / 0.236 | | | | 0.046 / 0.311 | | | |
