# Supplementary material for: Dealing with uncertainty: A high-density EEG investigation on how intolerance of uncertainty affects emotional predictions
Source: PLoS One. 2021 Jul 1;16(7):e0254045. doi: 10.1371/journal.pone.0254045 (PMC8248604; doi:10.1371/journal.pone.0254045)
Supplement: S4 Table — Dependent variables: lCNV and late l-ACC, l-SMA and l-dPCC. (DOCX) [file pone.0254045.s005.docx]

|  | **lCNV** | | | | **late l-ACC** | | | | **late l-SMA** | | | | **late l-dPCC** | | | |
| --- | --- | --- | --- | --- | --- | --- | --- | --- | --- | --- | --- | --- | --- | --- | --- | --- |
| *Predictors* | *Estimates* | *std. Error* | *CI* | *p* | *Estimates* | *std. Error* | *CI* | *p* | *Estimates* | *std. Error* | *CI* | *p* | *Estimates* | *std. Error* | *CI* | *p* |
| (Intercept) | -0.10 | 0.77 | -1.62 – 1.42 | 0.898 | 1.63 | 0.31 | 1.03 – 2.24 | **<0.001** | 1.52 | 0.28 | 0.96 – 2.08 | **<0.001** | 1.38 | 0.30 | 0.79 – 1.97 | **<0.001** |
| block50 | 0.48 | 1.04 | -1.57 – 2.52 | 0.646 | 0.24 | 0.37 | -0.49 – 0.96 | 0.519 | 0.30 | 0.35 | -0.39 – 0.99 | 0.391 | -0.16 | 0.36 | -0.86 – 0.54 | 0.655 |
| block50 × IUS | -0.02 | 0.04 | -0.09 – 0.05 | 0.635 | -0.00 | 0.01 | -0.03 – 0.02 | 0.807 | -0.01 | 0.01 | -0.03 – 0.02 | 0.664 | 0.01 | 0.01 | -0.02 – 0.03 | 0.518 |
| block50 × valenceneg | -1.23 | 1.47 | -4.12 – 1.66 | 0.401 | 0.31 | 0.52 | -0.71 – 1.34 | 0.549 | -0.25 | 0.50 | -1.22 – 0.73 | 0.622 | 0.64 | 0.51 | -0.36 – 1.64 | 0.207 |
| block50 × valenceneg × IUS | 0.02 | 0.05 | -0.08 – 0.12 | 0.706 | -0.01 | 0.02 | -0.05 – 0.02 | 0.491 | 0.00 | 0.02 | -0.03 – 0.04 | 0.882 | -0.02 | 0.02 | -0.05 – 0.01 | 0.233 |
| block50 × valencepos | -1.01 | 1.47 | -3.90 – 1.88 | 0.492 | -0.22 | 0.52 | -1.25 – 0.80 | 0.667 | 0.49 | 0.50 | -0.49 – 1.47 | 0.326 | 0.08 | 0.51 | -0.92 – 1.07 | 0.878 |
| block50 × valencepos × IUS | 0.01 | 0.05 | -0.08 – 0.11 | 0.793 | 0.01 | 0.02 | -0.03 – 0.04 | 0.609 | -0.02 | 0.02 | -0.05 – 0.01 | 0.210 | -0.00 | 0.02 | -0.04 – 0.03 | 0.886 |
| block75 | 2.73 | 1.04 | 0.68 – 4.77 | **0.009** | 0.24 | 0.37 | -0.49 – 0.96 | 0.517 | 0.15 | 0.35 | -0.55 – 0.84 | 0.677 | 0.45 | 0.36 | -0.26 – 1.15 | 0.212 |
| block75 × IUS | -0.09 | 0.04 | -0.16 – -0.02 | **0.010** | -0.01 | 0.01 | -0.03 – 0.02 | 0.648 | -0.00 | 0.01 | -0.03 – 0.02 | 0.820 | -0.01 | 0.01 | -0.04 – 0.01 | 0.279 |
| block75 × valenceneg | -2.60 | 1.47 | -5.49 – 0.29 | 0.078 | 0.00 | 0.52 | -1.02 – 1.03 | 0.994 | -0.01 | 0.50 | -0.99 – 0.97 | 0.986 | -0.14 | 0.51 | -1.13 – 0.86 | 0.789 |
| block75 × valenceneg × IUS | 0.08 | 0.05 | -0.02 – 0.18 | 0.111 | 0.00 | 0.02 | -0.03 – 0.03 | 0.995 | -0.00 | 0.02 | -0.03 – 0.03 | 0.980 | 0.00 | 0.02 | -0.03 – 0.04 | 0.839 |
| block75 × valencepos | -1.60 | 1.47 | -4.49 – 1.29 | 0.277 | 0.22 | 0.52 | -0.81 – 1.24 | 0.674 | 0.39 | 0.50 | -0.59 – 1.36 | 0.438 | -0.60 | 0.51 | -1.60 – 0.39 | 0.234 |
| block75 × valencepos × IUS | 0.05 | 0.05 | -0.05 – 0.15 | 0.331 | 0.00 | 0.02 | -0.03 – 0.04 | 0.990 | -0.02 | 0.02 | -0.05 – 0.02 | 0.291 | 0.02 | 0.02 | -0.01 – 0.06 | 0.213 |
| IUS | -0.01 | 0.03 | -0.06 – 0.04 | 0.762 | -0.01 | 0.01 | -0.03 – 0.01 | 0.177 | -0.01 | 0.01 | -0.03 – 0.01 | 0.223 | -0.01 | 0.01 | -0.03 – 0.01 | 0.249 |
| neu | *Reference* |  |  |  | *Reference* |  |  |  | *Reference* |  |  |  | *Reference* |  |  |  |
| valenceneg × IUS | -0.02 | 0.04 | -0.09 – 0.05 | 0.643 | 0.01 | 0.01 | -0.01 – 0.04 | 0.399 | 0.01 | 0.01 | -0.02 – 0.03 | 0.532 | 0.02 | 0.01 | -0.01 – 0.04 | 0.159 |
| pos | 0.39 | 1.04 | -1.65 – 2.43 | 0.707 | -0.12 | 0.37 | -0.85 – 0.60 | 0.738 | -0.39 | 0.35 | -1.08 – 0.30 | 0.271 | -0.00 | 0.36 | -0.71 – 0.70 | 0.990 |
| neg | 0.69 | 1.04 | -1.35 – 2.74 | 0.504 | -0.28 | 0.37 | -1.01 – 0.44 | 0.442 | -0.18 | 0.35 | -0.87 – 0.52 | 0.617 | -0.51 | 0.36 | -1.22 – 0.19 | 0.152 |
| valencepos × IUS | 0.00 | 0.04 | -0.07 – 0.07 | 0.914 | 0.00 | 0.01 | -0.02 – 0.03 | 0.943 | 0.01 | 0.01 | -0.01 – 0.04 | 0.210 | -0.00 | 0.01 | -0.02 – 0.02 | 0.973 |
| **Random Effects** | | | | | | | | | | | | | | | | |
| σ^2^ | 1.57 | | | | 0.20 | | | | 0.18 | | | | 0.19 | | | |
| τ_00_ | 0.17 _ID_ | | | | 0.08 _ID_ | | | | 0.05 _ID_ | | | | 0.07 _ID_ | | | |
| ICC | 0.10 | | | | 0.28 | | | | 0.23 | | | | 0.28 | | | |
| N | 36 _ID_ | | | | 36 _ID_ | | | | 36 _ID_ | | | | 36 _ID_ | | | |
| Observations | 324 | | | | 324 | | | | 324 | | | | 324 | | | |
| Marginal R^2^ / Conditional R^2^ | 0.101 / 0.187 | | | | 0.075 / 0.333 | | | | 0.060 / 0.276 | | | | 0.038 / 0.311 | | | |
