# Supplementary material for: Dealing with uncertainty: A high-density EEG investigation on how intolerance of uncertainty affects emotional predictions
Source: PLoS One. 2021 Jul 1;16(7):e0254045. doi: 10.1371/journal.pone.0254045 (PMC8248604; doi:10.1371/journal.pone.0254045)
Supplement: S5 Table — Dependent variables: P2 and bilateral TPJ. (DOCX) [file pone.0254045.s006.docx]

|  | **P2** | | | | **r-TPJ** | | | | **l-TPJ** | | | |
| --- | --- | --- | --- | --- | --- | --- | --- | --- | --- | --- | --- | --- |
| *Predictors* | *Estimates* | *std. Error* | *CI* | *p* | *Estimates* | *std. Error* | *CI* | *p* | *Estimates* | *std. Error* | *CI* | *p* |
| (Intercept) | 8.77 | 2.34 | 4.05 – 13.49 | **0.001** | 1.75 | 0.30 | 1.15 – 2.36 | **<0.001** | 1.41 | 0.32 | 0.78 – 2.04 | **<0.001** |
| block50 | -1.19 | 1.03 | -3.22 – 0.83 | 0.247 | 0.01 | 0.24 | -0.46 – 0.48 | 0.959 | 0.26 | 0.28 | -0.29 – 0.80 | 0.354 |
| block50 × IUS | 0.03 | 0.03 | -0.03 – 0.10 | 0.330 | 0.00 | 0.01 | -0.02 – 0.02 | 0.952 | -0.01 | 0.01 | -0.03 – 0.01 | 0.463 |
| block50 × valenceneg | 1.90 | 1.46 | -0.96 – 4.77 | 0.192 | -0.06 | 0.34 | -0.72 – 0.60 | 0.861 | -0.49 | 0.39 | -1.26 – 0.27 | 0.208 |
| block50 × valenceneg × IUS | -0.06 | 0.05 | -0.16 – 0.04 | 0.217 | 0.00 | 0.01 | -0.02 – 0.02 | 0.895 | 0.02 | 0.01 | -0.01 – 0.04 | 0.204 |
| block50 × valencepos | 2.63 | 1.46 | -0.24 – 5.49 | 0.072 | 0.18 | 0.34 | -0.49 – 0.84 | 0.601 | 0.48 | 0.39 | -0.28 – 1.25 | 0.215 |
| block50 × valencepos × IUS | -0.08 | 0.05 | -0.18 – 0.01 | 0.087 | -0.00 | 0.01 | -0.03 – 0.02 | 0.725 | -0.02 | 0.01 | -0.04 – 0.01 | 0.205 |
| block75 | 1.08 | 1.03 | -0.95 – 3.10 | 0.296 | 0.18 | 0.24 | -0.28 – 0.65 | 0.440 | 0.28 | 0.28 | -0.26 – 0.82 | 0.310 |
| block75 × IUS | -0.03 | 0.03 | -0.10 – 0.04 | 0.350 | -0.00 | 0.01 | -0.02 – 0.01 | 0.692 | -0.01 | 0.01 | -0.02 – 0.01 | 0.584 |
| block75 × valenceneg | 0.26 | 1.46 | -2.60 – 3.13 | 0.856 | 0.02 | 0.34 | -0.64 – 0.68 | 0.951 | -0.13 | 0.39 | -0.90 – 0.64 | 0.739 |
| block75 × valenceneg × IUS | -0.01 | 0.05 | -0.10 – 0.09 | 0.880 | -0.00 | 0.01 | -0.02 – 0.02 | 0.979 | 0.00 | 0.01 | -0.03 – 0.03 | 0.991 |
| block75 × valencepos | 1.33 | 1.46 | -1.53 – 4.20 | 0.360 | 0.30 | 0.34 | -0.37 – 0.96 | 0.380 | 0.53 | 0.39 | -0.24 – 1.30 | 0.174 |
| block75 × valencepos × IUS | -0.06 | 0.05 | -0.16 – 0.04 | 0.232 | -0.02 | 0.01 | -0.04 – 0.01 | 0.177 | -0.02 | 0.01 | -0.05 – 0.01 | 0.141 |
| IUS | -0.01 | 0.08 | -0.17 – 0.15 | 0.933 | 0.00 | 0.01 | -0.02 – 0.02 | 0.956 | 0.01 | 0.01 | -0.01 – 0.03 | 0.272 |
| neu | *Reference* |  |  |  | *Reference* |  |  |  | *Reference* |  |  |  |
| valenceneg × IUS | 0.01 | 0.03 | -0.06 – 0.07 | 0.875 | 0.00 | 0.01 | -0.02 – 0.02 | 0.919 | -0.01 | 0.01 | -0.03 – 0.01 | 0.249 |
| pos | -0.65 | 1.03 | -2.67 – 1.38 | 0.531 | -0.15 | 0.24 | -0.62 – 0.31 | 0.516 | -0.15 | 0.28 | -0.69 – 0.39 | 0.583 |
| neg | -1.28 | 1.03 | -3.30 – 0.75 | 0.216 | -0.15 | 0.24 | -0.61 – 0.32 | 0.537 | 0.23 | 0.28 | -0.31 – 0.77 | 0.409 |
| valencepos × IUS | -0.01 | 0.03 | -0.08 – 0.06 | 0.764 | -0.00 | 0.01 | -0.02 – 0.02 | 0.977 | 0.00 | 0.01 | -0.02 – 0.02 | 0.936 |
| **Random Effects** | | | | | | | | | | | | |
| σ^2^ | 1.55 | | | | 0.08 | | | | 0.11 | | | |
| τ_00_ | 14.39 _ID_ | | | | 0.18 _ID_ | | | | 0.18 _ID_ | | | |
| ICC | 0.90 | | | | 0.69 | | | | 0.62 | | | |
| N | 36 _ID_ | | | | 36 _ID_ | | | | 36 _ID_ | | | |
| Observations | 324 | | | | 324 | | | | 324 | | | |
| Marginal R^2^ / Conditional R^2^ | 0.025 / 0.905 | | | | 0.041 / 0.702 | | | | 0.038 / 0.636 | | | |
