# Supplementary material for: Dealing with uncertainty: A high-density EEG investigation on how intolerance of uncertainty affects emotional predictions
Source: PLoS One. 2021 Jul 1;16(7):e0254045. doi: 10.1371/journal.pone.0254045 (PMC8248604; doi:10.1371/journal.pone.0254045)
Supplement: S6 Table — Dependent variables: eLPP and early r-OFC and r-temporal pole. (DOCX) [file pone.0254045.s007.docx]

|  | **eLPP** | | | | **early r-OFC** | | | | **early r-temporal pole** | | | |
| --- | --- | --- | --- | --- | --- | --- | --- | --- | --- | --- | --- | --- |
| *Predictors* | *Estimates* | *std. Error* | *CI* | *p* | *Estimates* | *std. Error* | *CI* | *p* | *Estimates* | *std. Error* | *CI* | *p* |
| (Intercept) | 6.33 | 2.20 | 1.90 – 10.75 | **0.006** | 1.25 | 0.29 | 0.68 – 1.83 | **<0.001** | 1.48 | 0.33 | 0.83 – 2.14 | **<0.001** |
| block50 | -1.00 | 1.12 | -3.20 – 1.19 | 0.369 | 0.13 | 0.27 | -0.41 – 0.66 | 0.639 | 0.32 | 0.29 | -0.26 – 0.89 | 0.278 |
| block50 × IUS | 0.03 | 0.04 | -0.04 – 0.11 | 0.390 | -0.00 | 0.01 | -0.02 – 0.02 | 0.872 | -0.01 | 0.01 | -0.03 – 0.01 | 0.507 |
| block50 × valenceneg | 1.74 | 1.58 | -1.37 – 4.85 | 0.271 | -0.34 | 0.38 | -1.09 – 0.41 | 0.372 | -0.54 | 0.41 | -1.34 – 0.27 | 0.193 |
| block50 × valenceneg × IUS | -0.07 | 0.05 | -0.17 – 0.04 | 0.197 | 0.01 | 0.01 | -0.02 – 0.03 | 0.590 | 0.01 | 0.01 | -0.02 – 0.04 | 0.391 |
| block50 × valencepos | 1.79 | 1.58 | -1.32 – 4.90 | 0.258 | -0.43 | 0.38 | -1.18 – 0.32 | 0.261 | -0.61 | 0.41 | -1.42 – 0.20 | 0.138 |
| block50 × valencepos × IUS | -0.07 | 0.05 | -0.18 – 0.03 | 0.185 | 0.01 | 0.01 | -0.02 – 0.04 | 0.437 | 0.01 | 0.01 | -0.01 – 0.04 | 0.287 |
| block75 | 1.56 | 1.12 | -0.64 – 3.76 | 0.163 | 0.19 | 0.27 | -0.35 – 0.72 | 0.492 | 0.29 | 0.29 | -0.28 – 0.86 | 0.324 |
| block75 × IUS | -0.06 | 0.04 | -0.14 – 0.01 | 0.090 | -0.00 | 0.01 | -0.02 – 0.01 | 0.655 | -0.01 | 0.01 | -0.02 – 0.01 | 0.608 |
| block75 × valenceneg | 0.11 | 1.58 | -3.00 – 3.21 | 0.947 | 0.11 | 0.38 | -0.64 – 0.86 | 0.773 | 0.21 | 0.41 | -0.60 – 1.02 | 0.613 |
| block75 × valenceneg × IUS | -0.00 | 0.05 | -0.11 – 0.10 | 0.958 | -0.01 | 0.01 | -0.03 – 0.02 | 0.654 | -0.01 | 0.01 | -0.04 – 0.02 | 0.420 |
| block75 × valencepos | 0.08 | 1.58 | -3.03 – 3.19 | 0.960 | -0.34 | 0.38 | -1.09 – 0.41 | 0.374 | -0.60 | 0.41 | -1.41 – 0.21 | 0.146 |
| block75 × valencepos × IUS | 0.00 | 0.05 | -0.10 – 0.11 | 0.931 | 0.01 | 0.01 | -0.02 – 0.03 | 0.522 | 0.02 | 0.01 | -0.01 – 0.04 | 0.237 |
| IUS | -0.00 | 0.07 | -0.15 – 0.15 | 0.967 | 0.01 | 0.01 | -0.01 – 0.03 | 0.524 | -0.00 | 0.01 | -0.02 – 0.02 | 0.806 |
| neu | *Reference* |  |  |  | *Reference* |  |  |  | *Reference* |  |  |  |
| valenceneg × IUS | -0.00 | 0.04 | -0.08 – 0.07 | 0.929 | -0.02 | 0.01 | -0.04 – 0.00 | 0.053 | -0.00 | 0.01 | -0.02 – 0.02 | 0.717 |
| pos | 2.84 | 1.12 | 0.64 – 5.03 | **0.012** | 1.19 | 0.27 | 0.66 – 1.72 | **<0.001** | 0.85 | 0.29 | 0.27 – 1.42 | **0.004** |
| neg | 2.43 | 1.12 | 0.23 – 4.62 | **0.031** | 0.98 | 0.27 | 0.45 – 1.51 | **<0.001** | 0.56 | 0.29 | -0.01 – 1.13 | 0.055 |
| valencepos × IUS | -0.02 | 0.04 | -0.10 – 0.05 | 0.566 | -0.03 | 0.01 | -0.04 – -0.01 | **0.004** | -0.02 | 0.01 | -0.04 – 0.00 | 0.086 |
| **Random Effects** | | | | | | | | | | | | |
| σ^2^ | 1.82 | | | | 0.11 | | | | 0.12 | | | |
| τ_00_ | 12.27 _ID_ | | | | 0.14 _ID_ | | | | 0.19 _ID_ | | | |
| ICC | 0.87 | | | | 0.57 | | | | 0.61 | | | |
| N | 36 _ID_ | | | | 36 _ID_ | | | | 36 _ID_ | | | |
| Observations | 324 | | | | 324 | | | | 324 | | | |
| Marginal R^2^ / Conditional R^2^ | 0.086 / 0.882 | | | | 0.157 / 0.636 | | | | 0.100 / 0.649 | | | |
