# Supplementary material for: Dealing with uncertainty: A high-density EEG investigation on how intolerance of uncertainty affects emotional predictions
Source: PLoS One. 2021 Jul 1;16(7):e0254045. doi: 10.1371/journal.pone.0254045 (PMC8248604; doi:10.1371/journal.pone.0254045)
Supplement: S7 Table — Dependent variables: lLPP and late r-OFC and r-temporal pole. (DOCX) [file pone.0254045.s008.docx]

|  | **lLPP** | | | | **late r-OFC** | | | | **late r-temporal pole** | | | |
| --- | --- | --- | --- | --- | --- | --- | --- | --- | --- | --- | --- | --- |
| *Predictors* | *Estimates* | *std. Error* | *CI* | *p* | *Estimates* | *std. Error* | *CI* | *p* | *Estimates* | *std. Error* | *CI* | *p* |
| (Intercept) | 3.82 | 1.93 | -0.07 – 7.71 | 0.054 | 1.08 | 0.29 | 0.52 – 1.65 | **<0.001** | 1.18 | 0.31 | 0.57 – 1.80 | **<0.001** |
| block50 | -0.06 | 1.14 | -2.30 – 2.18 | 0.959 | 0.12 | 0.31 | -0.50 – 0.73 | 0.706 | 0.25 | 0.32 | -0.39 – 0.89 | 0.445 |
| block50 × IUS | 0.02 | 0.04 | -0.06 – 0.09 | 0.667 | -0.00 | 0.01 | -0.02 – 0.02 | 0.923 | -0.01 | 0.01 | -0.03 – 0.02 | 0.634 |
| block50 × valenceneg | 0.41 | 1.61 | -2.76 – 3.59 | 0.798 | -0.38 | 0.44 | -1.25 – 0.48 | 0.384 | -0.29 | 0.46 | -1.19 – 0.62 | 0.530 |
| block50 × valenceneg × IUS | -0.04 | 0.05 | -0.14 – 0.07 | 0.497 | 0.01 | 0.01 | -0.02 – 0.04 | 0.619 | 0.00 | 0.02 | -0.03 – 0.03 | 0.831 |
| block50 × valencepos | 0.02 | 1.61 | -3.15 – 3.19 | 0.990 | -0.70 | 0.44 | -1.57 – 0.17 | 0.113 | -0.95 | 0.46 | -1.85 – -0.04 | **0.041** |
| block50 × valencepos × IUS | -0.03 | 0.05 | -0.13 – 0.08 | 0.630 | 0.02 | 0.01 | -0.01 – 0.05 | 0.189 | 0.03 | 0.02 | -0.00 – 0.06 | 0.085 |
| block75 | 1.79 | 1.14 | -0.46 – 4.03 | 0.118 | 0.16 | 0.31 | -0.45 – 0.78 | 0.599 | 0.38 | 0.32 | -0.26 – 1.02 | 0.242 |
| block75 × IUS | -0.06 | 0.04 | -0.13 – 0.02 | 0.139 | -0.00 | 0.01 | -0.02 – 0.02 | 0.789 | -0.01 | 0.01 | -0.03 – 0.01 | 0.490 |
| block75 × valenceneg | -1.54 | 1.61 | -4.71 – 1.63 | 0.340 | -0.11 | 0.44 | -0.98 – 0.75 | 0.796 | 0.10 | 0.46 | -0.80 – 1.01 | 0.827 |
| block75 × valenceneg × IUS | 0.04 | 0.05 | -0.06 – 0.15 | 0.427 | 0.00 | 0.01 | -0.03 – 0.03 | 0.969 | -0.01 | 0.02 | -0.04 – 0.02 | 0.538 |
| block75 × valencepos | -0.97 | 1.61 | -4.15 – 2.20 | 0.547 | -0.60 | 0.44 | -1.47 – 0.27 | 0.174 | -1.02 | 0.46 | -1.93 – -0.12 | **0.027** |
| block75 × valencepos × IUS | 0.03 | 0.05 | -0.08 – 0.14 | 0.578 | 0.02 | 0.01 | -0.01 – 0.05 | 0.247 | 0.03 | 0.02 | -0.00 – 0.06 | 0.057 |
| IUS | 0.01 | 0.07 | -0.12 – 0.14 | 0.864 | 0.01 | 0.01 | -0.01 – 0.03 | 0.444 | 0.00 | 0.01 | -0.02 – 0.02 | 0.774 |
| neu | *Reference* |  |  |  | *Reference* |  |  |  | *Reference* |  |  |  |
| valenceneg × IUS | -0.01 | 0.04 | -0.09 – 0.06 | 0.758 | -0.02 | 0.01 | -0.04 – -0.00 | **0.043** | -0.01 | 0.01 | -0.03 – 0.01 | 0.467 |
| pos | 3.42 | 1.14 | 1.18 – 5.67 | **0.003** | 1.34 | 0.31 | 0.73 – 1.95 | **<0.001** | 1.33 | 0.32 | 0.69 – 1.97 | **<0.001** |
| neg | 3.06 | 1.14 | 0.82 – 5.31 | **0.008** | 1.18 | 0.31 | 0.56 – 1.79 | **<0.001** | 0.80 | 0.32 | 0.16 – 1.44 | **0.015** |
| valencepos × IUS | -0.04 | 0.04 | -0.12 – 0.03 | 0.262 | -0.03 | 0.01 | -0.05 – -0.01 | **0.003** | -0.03 | 0.01 | -0.06 – -0.01 | **0.003** |
| **Random Effects** | | | | | | | | | | | | |
| σ^2^ | 1.90 | | | | 0.14 | | | | 0.15 | | | |
| τ_00_ | 9.04 _ID_ | | | | 0.10 _ID_ | | | | 0.13 _ID_ | | | |
| ICC | 0.83 | | | | 0.40 | | | | 0.45 | | | |
| N | 36 _ID_ | | | | 36 _ID_ | | | | 36 _ID_ | | | |
| Observations | 324 | | | | 324 | | | | 324 | | | |
| Marginal R^2^ / Conditional R^2^ | 0.098 / 0.843 | | | | 0.184 / 0.514 | | | | 0.149 / 0.536 | | | |
