# Supplementary material for: Dealing with uncertainty: A high-density EEG investigation on how intolerance of uncertainty affects emotional predictions
Source: PLoS One. 2021 Jul 1;16(7):e0254045. doi: 10.1371/journal.pone.0254045 (PMC8248604; doi:10.1371/journal.pone.0254045)
Supplement: S1 File — Stimulus material: NimStim and IAPS picture numbers employed in the experimental paradigm. Data analysis: Planned planned paired-wise comparisons performed in the ERPs permutation analysis. (PDF) [file pone.0254045.s009.pdf]

Stimulus material

The NimStim model numbers were as follows: 1, 3, 6, 9, 28, 33, 34, and 36.

The IAPS picture numbers were as follows: 3000, 3010, 3015, 3030, 3051, 3053, 3060, 3068, 3071, 3080, 3100, 3102, 3110, 3120, 3130, 3140, 3150, 3400, 3550, 4647, 4651, 4652, 4653, 4656, 4658, 4659, 4664, 4666, 4669, 4670, 4672, 4680, 4683, 4687, 4690, 4694, 4695, 4800, 4810, 5621, 6190, 6200, 6210, 6211, 6213, 6230, 6242, 6243, 6250, 6260, 6300, 6312, 6313, 6315, 6350, 6360, 6510, 6530, 6540, 6550, 7000, 7002, 7004, 7006, 7009, 7010, 7020, 7025, 7030, 7031, 7034, 7035, 7036, 7037, 7039, 7040, 7041, 7050, 7052, 7056, 7060, 7080, 7090, 7100, 7110, 7130, 7140, 7150, 7170, 7175, 7211, 7217, 7224, 7233, 7234, 7235, 7491, 7495, 7500, 7510, 8021, 8030, 8031, 8034, 8040, 8080, 8160, 8161, 8178, 8179, 8180, 8185, 8186, 8193, 8200, 8210, 8370, 8400, 8490, and 9405.

Data analysis – planned comparisons

To test for the presence of overall predictive effects, we performed the paired-wise comparisons *100%* vs. *75%*, *100%* vs. *50%*, *50%* vs. *75%* blocks, collapsing emotional valence across blocks. In order to assess the interaction effect between S1/S2 valence and blocks, the contrasts *POS* vs. *NEU*, *NEG* vs. *NEU*, and *NEG* vs. *POS* were performed separately per blocks (*100%*, *75%, 50%*). Furthermore, for both S1- and S2-ERPs, difference waves were computed (*POS-NEU*, *NEG-NEU*, and *NEG-POS*) and compared between blocks.
